# Investigation of the Main Performance Indicators of Thermal-Insulating Dry-Mix Mortars Based on Shell Limestone Filler and Containing Hollow Glass Microspheres

**DOI:** 10.3390/ma19204322

**Published:** 2026-10-14

**Authors:** Yelzhan S. Orynbekov, Zaure N. Altayeva, Ruslan E. Nurlybayev, Axaya S. Yestemessova, Aktota A. Murzagulova, Alinur A. Iskakov, Gaukhar K. Abisheva, Abzal N. Alikhan, Indira B. Tashmukhanbetova

**Affiliations:** 1 Satbayev University, Almaty 050000, Kazakhstan; e.orynbekov@kazgasa.kz (Y.S.O.); aktoty_sun@mail.ru (A.A.M.); isk_888@mail.ru (A.A.I.); abisheva35234@gmail.com (G.K.A.); a.alikhan@satbayev.university (A.N.A.); 2 International Educational Corporation, Almaty 050043, Kazakhstan; axaya73@mail.ru

**Keywords:** hollow glass microspheres, amorphous aluminosilicates, shell limestone, thermal-insulating dry-mix mortar, thermal conductivity, complex modifier

## Abstract

This paper presents a study of thermal-insulating dry-mix mortars containing shell limestone, shell limestone extraction waste, and hollow microspheres. The effects of shell limestone and fillers on physical, mechanical, and thermal properties were investigated. The microstructure of shell limestone was examined by scanning electron microscopy (SEM) and energy-dispersive X-ray spectroscopy (EDS) using a JEOL JSM-7000F scanning electron microscope. The elemental mass fractions confirmed the carbonate nature of the shell limestone raw material (wt.%: O, 50.3–43.7; Ca, 34.1–44.2; C, 15.6–12.1). Finely dispersed fillers, hollow glass microspheres, and amorphous aluminosilicates were found to be suitable for producing a low-density thermal-insulating lime–shell limestone–cement composition. The particle size of the shell limestone powder was 5.67 × 10^−6^ m, with calcium carbonate (CaCO_3_) and silica (SiO_2_) contents of 71.0% and 22.5%, respectively. The shell limestone and microsphere proportions in the binder substantially affected thermal conductivity (0.090–0.113 W/(m·°C)), compressive strength (3.25–4.05 MPa), adhesion strength to the substrate (0.8–1.2 MPa), flexural tensile strength (2.0–2.5 MPa), and water absorption coefficient (0.3–0.5%). The optimum thermal-insulating dry-mix formulation contained, by mass: lime, 25–40%; shell limestone, 15–20%; glass microspheres, 15–20%; amorphous aluminosilicates, 5–10%; diatomite, 2–10%; and cement, 0.5–4.0%.

## 1. Introduction

In today’s rapidly developing world, environmental awareness is increasing, while infrastructure and building owners and developers face major challenges in creating a sustainable future while maintaining cost efficiency. One solution is to introduce a comprehensive procurement system for environmentally friendly construction materials. Construction materials have long been considered difficult to decarbonize, particularly in terms of reducing carbon dioxide emissions associated with their production and use.

Environmentally friendly construction materials prioritize sustainability throughout their life cycle, with lower CO_2_ emissions during production, construction, operation, and disposal than conventional materials. Energy efficiency is one approach to minimizing the direct and indirect emissions associated with the production of environmentally friendly materials.

Large-scale mineral resource consumption and environmental pollution adversely affect the planet. Developed countries and Kazakhstan have set global goals to reduce atmospheric CO_2_ emissions and support sustainable development.

The carbon footprint, comprising greenhouse gas emissions resulting from human activity that adversely affect the urban climate, is becoming a principal component of the ecological footprint. It can be reduced through responsible use of natural resources and energy and the development of environmentally safe urban architectural spaces.

The increasing pace of modern construction requires new ideas, developments, and technologies. Rising energy tariffs also make thermal insulation materials increasingly important [1,2,3,4,5,6,7]. A shortage of effective thermal insulation materials leads to substantial heat losses [8,9,10].

The thermal insulation market offers a wide variety of materials differing in raw material type, shape, structure, and technical properties. Mineral wool and expanded polystyrene are among the most widely used materials [11,12,13,14,15].

Conventional thermal insulation materials share several disadvantages: they are inefficient for insulating complex surfaces, do not completely eliminate thermal bridges, and require additional anticorrosion materials when used on metal structures. Expanded polystyrene boards with a density of 40 kg/m^3^ have a dry thermal conductivity of 0.038 W/(m °C), increasing to 0.05 W/(m °C) in building envelopes when wall moisture during operation is considered [5,16,17,18].

Developing thermal-insulating coating formulations based on hollow microspheres and porous mineral materials is one of the most promising ways to improve the thermal insulation of building envelopes [19,20,21,22,23].

A thermal-insulating dry-mix wall coating based on hydrated lime, diatomite, and a modifying additive was developed in [24]. Its thermal conductivity was 0.16 W/(m °C), adhesion strength was 0.6–0.9 MPa, and compressive strength was 3–4 MPa. Dry-mix consumption was 0.6–0.8 kg/m^2^ for a 5 mm finishing layer.

Suitable dry thermal-insulating plaster formulations based on cement–perlite compositions with modifying additives were developed by considering the rheology of cement–perlite systems and the physical and mechanical properties of hardened thermal-insulating plaster mortars [25]. The kinetics of structure formation in cement and cement–perlite compositions containing modifying additives were identified. Varying the mixture composition and additive dosages was found to regulate the structural and mechanical properties of mortars and enable materials with the required processing and technical characteristics to be produced.

The optimum structure for thermal insulation materials maximizes the filling of the composite with lightweight amorphous solid particles. To eliminate the convective component of heat transfer, the trapped-air cells must be substantially smaller than 2 mm [26].

A method for producing porous aggregate was developed in [27]. To reduce aggregate bulk density and water saturation, dried granules were crushed, moistened with sulfite–alcohol or sulfite–yeast liquor, granulated, and dried. Fly ash from thermal power plants containing more than 10% unburned carbon was used as the raw material.

A porous glass thermal insulation material with a bulk density of 100–150 kg/m^3^ was developed in [28]. Its production involved preparing a mixture of sodium silicate solution and processing additives, partially dehydrating the mixture, dispersing or granulating it, and expanding the granules. The raw materials were sodium silicate solution, mineral fillers, and additives. Finely ground fillers included limestone, sand, talc, kaolin, tripoli, perlite, and thermal power plant ash.

Large quantities of finely dispersed mineral fillers obtained from sedimentary rocks are used to manufacture thermal insulation materials. Heat transfer in these materials occurs through conduction along individual particles, heat transfer and conduction at particle contacts, molecular conduction through the medium filling the interparticle spaces, heat transfer at the medium–surface interface, gas and moisture convection, and radiation between particles [29].

The self-organization of dispersed powders plays an important role in forming a “structure within a structure”, creating a multilevel pore system. This reduces the conductive and radiative components of effective thermal conductivity by decreasing the volume fraction of highly conductive grains and reflecting and refracting electromagnetic waves at structural heterogeneities [30].

Diatomite, tripoli, and zeolite are particularly effective in terms of thermal insulation because their particles have an intrinsic nanoscale pore structure [31,32].

Its siliceous, finely dispersed composition and large specific surface area give diatomite excellent thermal-insulating, abrasive, acid-resistant, and refractory properties [27], making it suitable for thin-film thermal-insulating coating formulations.

Factors affecting the physical and mechanical properties of mortar based on lime obtained from marine shells were investigated in [33]. The study considered shell calcination temperature, lime–sand–water ratios, and an organic additive, sticky rice, in relation to hydration, structure formation, compressive and flexural strength, water resistance, and durability. The potential of shell-derived lime as a sustainable binder for restoration and construction mortars was assessed. The results demonstrated practical value for developing modern lime-based and thermal-insulating dry-mix mortars and restoration plasters.

The effects of hydrated lime and cellulose ether on cement plastering mortars were studied in [34], including water retention, rheology, workability, adhesion, application characteristics, and service performance. Their combined action in dry-mix mortar development was assessed. The effects on selected paste and plaster properties were also evaluated using the Box–Behnken statistical method.

The effects of cellulose ether on setting and structure formation in cement and cement–lime dry-mix composites were investigated in [35]. The study examined cement hydration kinetics, initial and final setting times, water retention, cement paste structure, and the processing properties of modified mortars.

The effects of limestone powder, redispersible polymer powder (RDP), and cellulose ether on cement paste rheology and early structure formation were studied in [36]. Combined modifying additives were assessed in terms of viscosity, flowability, water retention, cement hydration, microstructure formation, and component interactions during early hardening. The effectiveness of combining mineral and polymer modifiers in modern dry-mix mortars was established.

Limestone and shell-derived lime as fine aggregate replacements in lime plaster were investigated in [37]. The effects of mortar composition on physical and mechanical properties, microstructure, strength, porosity, compatibility with historic building materials, and durability were considered. The suitability of natural calcareous materials for restoration plaster coatings with improved service properties was assessed.

The mechanical, thermal-insulating, and acoustic properties of an innovative lime–wool composite intended as a thermal insulation building material were reported in [38]. The effects of natural wool filler on strength, density, thermal conductivity, sound absorption, and lime matrix structure were examined. The service properties of this environmentally friendly composite and its potential for energy-efficient thermal-insulating plaster and finishing systems were comprehensively assessed.

Innovative thermal-insulating renders based on natural hydraulic lime with expanded cork and crushed oyster shells as environmentally friendly aggregates were developed in [39]. The effects of replacing insulating aggregate with oyster shells on rheology, density, compressive strength, thermal conductivity, and service properties were examined. The feasibility of using biobased waste to create energy-efficient and environmentally sustainable dry-mix mortars consistent with circular economy principles was established.

Study [40] investigated the use of mussel-shell waste generated by the canning industry as a source of calcareous material for mortars. The shell-derived material consisted predominantly of calcium carbonate and was considered an alternative to conventional limestone raw materials. The authors examined the influence of this shell-derived limestone on mortar structure and properties, demonstrating the feasibility of recycling shell waste as a mineral component of construction mortar.

Article [41] investigated the use of common shell waste as an alternative to conventional limestone filler in cementitious materials. Different shell raw materials, including oyster and mussel shells, were ground to powder and compared with conventional limestone filler. In mortar formulations, 20% of the cementitious material by mass was replaced with shell powder or limestone filler. Fresh-mortar properties and hardened-material characteristics, including flowability and compressive strength, were evaluated.

Wang et al. [42] investigated thermal-insulating cementitious syntactic foams containing 0–56 vol.% hollow glass microspheres (HGM). Specimens were produced by a three-stage process, and thermal conductivity was measured by the transient plane-source method. Effective thermal conductivity decreased almost linearly with increasing HGM content; at 56 vol.% microspheres, the reduction exceeded 47% relative to the neat cementitious material. Numerical modeling that accounted for the random HGM distribution agreed well with the experimental results. The analysis indicated that the cementitious matrix governed effective thermal conductivity, whereas the microsphere wall and gas core made smaller contributions.

Aslani and Wang [43] aimed to produce high-performance lightweight engineered cementitious composites (LWECC) using HGM as an ultra-lightweight filler. Three microsphere types with densities of 0.25, 0.40, and 0.60 g/cm^3^ were used to replace 40% and 60% of the fly ash mass in the control mixture. Mechanical properties were measured after 7 and 28 days of curing, and SEM, EDS, and morphological analysis of filler distribution were also performed. The authors identified HGM, which contains a substantial proportion of recycled glass, as a promising component for lightweight strain-hardening composites.

Lee et al. [44] addressed the simultaneous achievement of high strength and low density. To prevent the floating of hollow particles, the authors used a low water-to-cement ratio, a polycarboxylate superplasticizer, and viscosity-enhancing fillers. The resulting composites had densities below 1.5 g/cm^3^ and 28-day compressive strengths of 69 MPa after normal curing and 97 MPa after heat treatment. The study also showed that the hollow particles did not actively participate in hydration and acted mainly as an ultra-lightweight inert filler.

Gómez Nemocón et al. [45] investigated the partial replacement of cement with hollow glass microspheres in self-compacting concrete at dosages of 0% and 5%. The main focus was the rheological effect of HGM associated with their spherical shape. Properties were assessed by direct measurements using a rotational rheometer and by indirect slump-flow, J-ring, and L-box tests; the data were analyzed using the Bingham and modified Bingham models. HGM improved fresh-state mixture performance but slightly reduced hardened-state properties.

The international literature demonstrates the multifaceted relevance of finely dispersed mineral fillers for thermal-insulating coatings. It provides a basis for further research and development of dry-mix thermal insulation technologies using local mineral resources, including shell limestone and extraction waste available in large quantities in Kazakhstan’s Mangystau Region.

The aim of this study is to develop a production technology and optimize thermal-insulating dry-mix mortar formulations based on shell limestone and hollow microspheres, both glass and aluminosilicate, with improved service and thermophysical properties.

The novelty of this study lies in the development of thermal-insulating dry-mix compositions that combine shell limestone from Kazakhstan’s Mangystau deposits with hollow glass microspheres to achieve low thermal conductivity. The study establishes effective component proportions and evaluates the resulting physical, mechanical, and thermophysical properties. The low water demand is attributed to the small proportion of open micropores and the spherical shape of the microspheres.

An analysis of the available literature on the physicochemical, physical, technical, and thermophysical properties of shell limestone-based dry-mix mortars established the main research direction. The materials investigated were binders (lime and cement), aggregates (sand and shell limestone), fillers (shell limestone powder, diatomite powder, and hollow microspheres), and chemical and polymer additives. Their structural relationships and effects on the principal service properties of thermal-insulating dry-mix mortars were investigated.

## 2. Materials and Methods

Particular attention was paid to developing effective thermal-insulating dry-mix mortars based on finely ground shell limestone and diatomite powders with hollow glass and aluminosilicate microspheres.

### 2.1. Materials

The components used to produce the thermal-insulating dry-mix mortars were a mineral binder, hollow aggregates, fine fillers, functional additives and modifiers, and tap water.

Shell limestone was obtained as waste from natural building-stone extraction at the Zhetybay deposit. It is an organogenic sedimentary rock consisting of small shell fragments cemented by an uneven, finely crystalline carbonate binder with a layered structure. Its advantages as a building material include environmental compatibility, high thermal insulation capacity compared with brick or concrete, and affordability.

Commercial 3M™ K15 hollow glass microspheres were used as an existing hollow filler; the present study did not investigate the synthesis of glass microspheres or the use of recycled glass. Before use, defective particles were removed by flotation in water. The chemical composition reported for the microspheres, including B_2_O_3_, is the product composition; the boron-oxide content was not varied as an experimental factor in this study. Typical microsphere diameters depend on the application and generally range from 10 to 200 µm, with shell wall thicknesses of 0.5–2.0 µm [46].

Amorphous aluminosilicates were prepared as described below and used as a fine mineral modifying additive to improve mortar properties. Their specific surface area was 689 m^2^/kg.

Diatomite is a loose or weakly cemented siliceous sedimentary rock composed predominantly of algal frustules and may be white, gray, or pinkish. It has high, uniformly distributed porosity of 80–85%. Silica is amorphous and accounts for 78–95%.

The diatomite was obtained from the Zhalpak deposit in the Mugalzhar District, Aktobe Region. Its mean lump density was 300–500 kg/m^3^, mean loose-state density was 220–350 kg/m^3^, true density was 2000 kg/m^3^, and bulk density was 280 kg/m^3^. The modified diatomite contained SiO_2_, 86.6%; Al_2_O_3_, 6.5%; and Fe_2_O_3_, 3.2%, with calcium, magnesium, and sulfur oxides each below 1%. Thus, the modified diatomite consisted of dispersed silicon dioxide particles with a developed nanoscale porous structure.

Lime supplied by PS Vostok Trade had an activity of 80–89%, a true density of 2260 kg/m^3^, a bulk density of 285 kg/m^3^, and a specific surface area of 11,000 cm^2^/g (Table 1).

**Table 1:** Main characteristics of lime.

Grade	CaO + MgO Activity, %	Active MgO, %	Unslaked Particles, %	Slaking Time, min	Slaking Temperature, °C
2	80–89 minimum	5 maximum	11 maximum	2–4 maximum	87–93 minimum

White Portland cement, grade PTs400D20(Italy), had an absolute-scale reflectance of 85% (Table 2).

**Table 2:** Chemical composition of white cement.

Oxides	CaO	SiO_2_	Al_2_O_3_	SO_3_	Fe_2_O_3_	MnO	CuO	TiO_2_	SrO	R_2_O	OH	Σ
Content, %	68.0	23.3	4.5	3.0	0.3	0.01	0.01	0.2	0.1	0.01	0.57	100

The sand had a fineness modulus of 2.75, belonged to the coarse group and class I, and complied with GOST 8736-2014 [47], “Sand for Construction Works. Specifications”.

Sika-3180, a polycarboxylate ether-based superplasticizer, was used in the dry mixes to maintain workability over an extended period.

The redispersible additive Neolit-4400 improves adhesion, elasticity, and crack resistance.

Zincum hydrophobic powder is based on zinc stearate and is intended to improve water impermeability.

### 2.2. Methods

The microstructure of shell limestone was investigated by scanning electron microscopy (SEM) and energy-dispersive X-ray spectroscopy (EDS) using a JEOL JSM-7000F scanning electron microscope.

The particle-size distribution of the fine filler, comprising shell limestone stone-cutting waste powder and limestone powder, was measured by the authors using a Microtrac S3500 diffraction analyzer with a measurement range of 0.021–2000 µm. Particles in the upper size range were measured dry, with the sample supplied in an air stream. Fine materials were measured wet, using water as the carrier medium. Ultrasonic dispersion was applied for 3 min to distribute the particles and prevent coagulation. Measurements used laser diffraction at a wavelength of 780 nm, with a relative measurement deviation not exceeding 0.7%.

Compressive strength was determined on at least three 70.7 × 70.7 × 70.7 mm cubes at ages of 3, 7, 14, and 28 days. Mortar with a consistency greater than 5 cm was placed in split molds without baseplates. The molds were placed on bricks with a moisture content not exceeding 2% and water absorption of 10–15%, covered beforehand with damp newspaper. Filled molds were stored before demolding at (20 ± 2) °C and (65 ± 10)% relative humidity. Specimens were demolded 24 h after casting and stored in the same room under the same conditions, avoiding drafts and proximity to heaters. At 3, 7, 14, and 28 days, the specimens were visually inspected, measured using a caliper with an accuracy of 0.1 mm, and placed in a testing press. The load was increased continuously at (0.6 ± 0.4) MPa/s until failure. Compressive strength R was calculated for each specimen to 0.01 MPa (0.1 kgf/cm^2^). Testing followed GOST 5802-2024 [48].

Plastic strength, or yield shear stress, of the finishing mortar was determined using a KP-3 cone plastometer. The method measures the penetration depth of a cone indenter into the composition under a static load. When the cone reaches equilibrium, the shear stress τ in the material equals the yield stress τ_0_.

Water retention was determined in accordance with the applicable standards. Ten sheets of blotting paper, each 150 × 150 mm, were weighed to 0.1 g and placed on a glass plate measuring 150 × 150 × 5 mm. A layer of gauze was placed over the paper, followed by a metal ring with an internal diameter of 100 mm and a height of 12 mm, and the assembly was reweighed. Thoroughly mixed mortar was placed level with the ring edge, weighed, and left for 10 min. The ring and mortar were then carefully removed with the gauze, and the blotting paper was weighed to 0.1 g. Water retention was determined from the percentage water content before and after testing. Each sample was tested twice; the result was the arithmetic mean of two measurements differing by no more than 20% of the lower value. Testing followed GOST 5802-2024 [48]. The volume of open capillary pores was estimated from volumetric water absorption using Equation (1):

(1) P_o_ = W_o_,

where

W_o_ is the volumetric water absorption of a specimen in the series, calculated using Equation (2):

(2) $$W_{o}=\frac{w_{m\cdot }\rho_{m}}{\rho_{\mathrm{water}}}$$

Thermal conductivity was measured by the authors using an ITP-MG4 instrument. A steady heat flow was established through a specimen of known thickness, perpendicular to its faces. Heat flux density, the temperatures of the opposite faces, and specimen thickness were measured. Testing followed GOST 7076-99 [49].

Shrinkage deformation was measured by the authors using an IZA-2 optical comparator.

The contact-zone frost resistance grade was considered compliant when, after the prescribed number of freeze–thaw cycles for the relevant mortar grade, the mean adhesion strength of the test specimens had decreased by no more than 20% relative to the mean strength of the control specimens.

Testing was stopped if intermediate measurements showed a decrease of more than 20% in mean adhesion strength relative to the controls.

Adhesion strength was determined by the normal pull-off method, based on the limiting stress at surface separation. Cylindrical dollies, 50 mm in diameter, were bonded to the finished surface using EDP epoxy adhesive. The specimen was positioned horizontally, a dynamometer was attached to the dolly, and the required detachment force was recorded. Bond strength between the coating and substrate was calculated using the formula in the applicable standard. Testing followed GOST 31356-2007 [50].

Frost resistance was determined using the basic repeated freeze–thaw method. For each composition, six control and twelve test cubes measuring 70.7 × 70.7 × 70.7 mm were tested in series at the specified age after conditioning at 20–23 °C and 50–60% relative humidity. Testing followed GOST 5802-2024 [48].

Contact-zone frost resistance was determined from the adhesion strength of the thermal-insulating mortar to concrete and cement–sand substrates using the dolly method.

Prismatic specimens measuring 50 × 50 × 10 mm were prepared. A stencil was placed on the substrate and filled with ready-to-use mortar of the specified consistency grade. The surface was leveled with a metal spatula and the stencil was immediately removed. During structure formation, before hardening began, truncated conical rings were pressed through the mortar layer to the substrate while rotating. The rings were then carefully withdrawn while continuing to rotate them. Spacing between rings and between rings and substrate edges was at least 50 mm.

Before testing, specimens were stored for 7 days at (20 ± 2) °C and (95 ± 5)% relative humidity, followed by 21 days at (20 ± 2) °C and (65 ± 5)% relative humidity. Total curing time was 28 days.

Fifteen specimens were prepared for each mortar: five controls and ten specimens for repeated freezing and thawing, comprising five for intermediate and five for final testing.

After 27 days, dollies were bonded to the hardened specimens using epoxy or another high-strength, rapid-curing adhesive and maintained for 24 h at (20 ± 2) °C and (65 ± 5)% relative humidity. The pull-off force was then measured using a press or another measuring device, applying force to the dolly at an increasing rate of 250 ± 50 m/s. Testing followed GOST 31356-2007.

Adhesion strength to the substrate was determined from the maximum force applied perpendicular to the specimen surface at which separation from the substrate occurred.

The adhesion strength of an individual specimen, in MPa, was calculated using Equation (3):

(3) $$A=\frac{F}{S}.$$

where:

F is the maximum force required to detach the specimen from the substrate, N;

S is the contact area between the specimen surface and the substrate, mm.

Each individual adhesion strength value was rounded to 0.1 MPa. The test result was the arithmetic mean for all specimens, also rounded to 0.1 MPa. Testing followed GOST 31356-2007.

## 3. Results

### 3.1. Development of Formulations and a Preparation Method for Thermal-Insulating Dry-Mix Mortars Based on Shell Limestone Filler and Hollow Glass Microspheres

The principal properties of the developed thermal-insulating mortars were investigated as follows. Samples were taken from at least three locations at different depths in the raw material and mixed for a further 30 s before testing. Mixing began when the entire dry sample came into contact with water. The preparation sequence was:

- Drying the raw components to constant mass at 70–100 °C;
- Mixing the mortar for 120 s;
- Stopping the mixer and removing material adhering to its walls for 90 s, followed by another 60 s of mixing. Total mixing time after water addition was at least 3 min, excluding the stoppage.

The thermal-insulating dry mix was prepared as a homogeneous mixture of hydrated lime, finely ground shell limestone powder from the Beineu deposit and its extraction waste, hollow glass microspheres, diatomite, an amorphous aluminosilicate mineral additive, sand, Sika-3180 superplasticizer, Neolit P4400 redispersible powder, Zincum-5 hydrophobic powder, and cement. Before use, water was added to obtain a readily workable plastic paste.

Additive formulations were selected and optimized by evaluating changes in the rheological and processing properties of the water-mixed thermal-insulating mortars. The modifying additive compositions are given in Table 3.

**Table 3:** Optimum modifying additive formulations for thermal-insulating dry-mix mortars.

Components	Component Content, wt.%		
	CM-1	CM-2	CM-3
Amorphous aluminosilicates	31.25	49.18	68.965
Diatomite	62.5	39.344	13.793
Sika-3180 superplasticizer	3.125	4.92	6.896
Zincum-5 hydrophobic powder	3.125	6.557	10.344

The study presents the results of optimizing a dry-mix mortar formulation containing a complex modifier.

Experimental investigations used mathematical experimental design methods. A second-order orthogonal design comprising 15 experiments was used. The variables were shell limestone (X_1_), glass microspheres (X_2_), and complex modifier (X_3_). Factor levels and the experimental design in coded values are given in Table 4 and Table 5.

**Table 4:** Levels of the variable factors.

Factors Investigated			Factor Levels		
Symbol	Name	Unit	−1	0	+1
X_1_	Shell limestone	% of mixture mass	15.0	17.25	20.0
X_2_	Glass microspheres	% of mixture mass	20.0	17.25	15.0
X_3_	Complex modifier	% of mixture mass	16	15.25	14.5

**Table 5:** Experimental design and results for thermal-insulating compositions with different fillers.

Mix No.	Factors in Coded Values			Results	
	Shell Limestone X_1_	Glass Microspheres X_2_	Complex Modifier X_3_	Density, kg/m^3^, Y_1_	Thermal Conductivity, W/(m K), Y_2_
1	0	1	1	850	0.183
2	0	1	−1	830	0.176
3	0	−1	1	800	0.168
4	0	−1	−1	780	0.166
5	1	−1	0	730	0.167
6	1	1	0	680	0.160
7	1	0	−1	650	0.145
8	1	0	1	600	0.140
9	1	0	0	610	0.143
10	0	0	0	630	0.148
11	−1	−1	0	580	0.113
12	−1	1	0	575	0.096
13	−1	0	−1	585	0.114
14	−1	0	1	570	0.112
15	−1	0	0	620	0.148

Particular attention was paid to developing a method for preparing effective thermal-insulating dry-mix mortars using finely dispersed shell limestone and diatomite fillers and hollow glass microspheres. The production process is shown in Figure 1.

The thermal-insulating dry mix was prepared using this method; its optimum composition is given in Table 6.

**Table 6:** Optimum thermal-insulating dry-mix mortar formulations.

Components	Component Content, wt.%	
	Control	Developed
Lime	22.89–39.89	25–40
Shell limestone powder	-	20–15
Hollow glass microspheres	-	15–20
Sand	20–15	10–15
Neolit P4400 redispersible powder		10–5
Redispersible polymer additive	1	-
Cement	5	4–0.5
Modified diatomite	51–39	-
Water-retaining additive	0.1	-
Air-entraining additive	0.01	-
Complex modifier CM		16–14.5

Because of the fineness of the components, a forced-action rotor mixer providing intensive mixing was used to produce the thermal-insulating dry mixes. The optimum mixing time was 5–6 min.

### Characterization Results for the Principal Components

Shell limestone extraction waste was obtained from part of the Zhetybay deposit in the Karakiya District of the Mangystau Region.

SEM analysis showed that the shell limestone extraction waste had a heterogeneous particulate structure with a developed rough surface. The micrographs show irregular angular particles, agglomerates, and intergranular voids of different sizes (Figure 2).

At ×500 magnification, the overall structural heterogeneity and the distribution of particles of different shapes and sizes are visible. At ×1000, plate-like structures, rough surface microrelief, and fine particles can be seen more clearly.

The porous structure and intergranular voids may reduce the thermal conductivity of the raw material by increasing the volume of air inclusions.

Energy-dispersive X-ray spectroscopy (EDS) was performed to determine the elemental composition of the raw material (Figure 3 and Table 7).

**Table 7:** EDS analysis of shell limestone extraction waste from the Zhetybay deposit [51].

Element	Mass Fraction, %	Atomic Fraction, %
C	15.61	24.56
O	50.25	59.34
Ca	34.14	16.09

EDS analysis identified oxygen (O), calcium (Ca), and carbon (C) as the main elements. The high calcium and oxygen contents indicate the predominance of carbonate compounds characteristic of shell limestone. The measured mass and atomic fractions confirmed the carbonate nature and mineral composition of the extraction waste (wt.%/at.%: O, 50.25/59.34; Ca, 34.14/16.09; C, 15.61/24.56).

The commercial 3M™ K15 hollow glass microspheres used in this study were separated from defective particles by flotation in water. This yielded microspheres measuring 20–100 µm, with a mean diameter of 75 µm and wall thicknesses of 1–2 µm (Figure 4).

The dried microspheres had a bulk density of 0.107 g/cm^3^ and a thermal conductivity of 0.05 W/(m K). Hollow glass microspheres act as a light-reflecting pigment with high hiding power, making them suitable for thermal-insulating compositions.

Microspheres can improve the thermal insulation and sound absorption of composite materials. Their main technical characteristics and chemical composition are given in Table 8 and Table 9.

**Table 8:** Main characteristics of 3M™ K15 hollow glass microspheres [51].

Grade	Wall Thickness, µm	ρ, g/cm^3^	Vinternal/Vtotal ratio	Crush Resistance, 90% Intact, MPa (Bar)	Thermal Conductivity, W/(m K)	Mean Diameter, µm
K15	0.61	0.154	0.942	2.14 (214)	0.0511	61

**Table 9:** Chemical composition of glass microspheres [51].

Oxides	Si_2_O	Na_2_O	CaO	B_2_O_3_
Content, %	78	5	10	4

The mineral portion of the dry mix comprised finely dispersed fillers of different origins and particle sizes.

The following mineral additives were used:

- Shell limestone powder, obtained by grinding a porous sedimentary calcareous rock composed mainly of marine shells and shell fragments. Its mean particle size was 5.67 × 10^−6^ m, with mean calcium carbonate (CaCO_3_) and silica (SiO_2_) contents of 71.0% and 22.5%, respectively. The particle-size distribution is shown in Figure 5.

- Diatomite powder, obtained by finely grinding a yellowish sedimentary rock from the Aktobe deposit, Kazakhstan, consisting predominantly of diatom frustules. Its mean particle size was 12.4 × 10^−6^ m. Mean contents were SiO_2_, 86.6%; Al_2_O_3_, 6.5%; and Fe_2_O_3_, 3.2%, with calcium, magnesium, and sulfur oxides each below 1%. Diatomite consists of dispersed silica particles with a developed nanoscale pore structure.

Mineralogical analysis showed that the principal diatomite phases included quartz, montmorillonite, muscovite, and kaolinite (Figure 6). Its chemical and mineralogical composition is given in Table 10.

The particle-size distribution is shown in Figure 7.

The amorphous aluminosilicate additive was prepared by precipitating aluminosilicates from sodium silicate solution with a silicate modulus of 2.81 using a 15% solution of technical-grade aluminum sulfate, Al_2_(SO_4_)_3_, followed by washing with distilled water. The precipitate was dried at 100 °C to constant mass and ground to a specific surface area of 689 m^2^/kg. Its physicochemical properties are presented in Table 11.

Sika-3180 is a polycarboxylate ether-based superplasticizer for dry mixes with extended workability retention, intended for low-heat mortars. Its main properties are given in Table 12.

The main properties of Neolit P4400 are given in Table 13. Polyvinyl alcohol is listed as the stabilization system of this commercial redispersible powder; it was not introduced as a separate experimental component, and its molecular mass was not an experimental parameter of the present study.

Zincum-5 is a zinc stearate-based hydrophobic powder. Its main properties are given in Table 14.

The composition and physical and technical properties of the principal components demonstrated their suitability for use in the developed thermal-insulating dry-mix mortars and provided a basis for evaluating the physical, mechanical, and thermophysical properties of the resulting materials.

### 3.2. Technological Aspects of Producing Thermal-Insulating Dry-Mix Mortars Based on Shell Limestone and Hollow Spheres

The main factor governing mortar workability and uniformity is the solid-to-liquid ratio in the lime–cement suspension, commonly expressed as the water-to-lime-plus-cement ratio, W/(L+C). Aggregate density and content also contribute. Other properties, including workability, have practical value only when mortar uniformity is ensured.

The Krieger–Dougherty equation [53] can be used to estimate the viscosity of the lime–cement–mineral suspension, Equation (4):

(4) $$\mu_{m}{\left(1-\frac{\nu }{\varrho }\right)}^{-\alpha \varrho },$$

where:

α = 2.5; ρ = ν_m_ = 0.59–0.65 [37] is the particle volume fraction ν at which viscosity tends to infinity (the most likely value for suspensions is ν_m_ = 0.61 ± 0.02).

The viscosity of the lime–cement suspension at different W/(L+C) ratios is given in Table 15.

Adding aggregate to mortar suspensions thickens the system by increasing the wetted surface area; workability therefore depends on dispersed-phase content. According to [54], the de Bruijn–Barger concentration relationship can describe the viscosity μd of a dispersed system, Equation (5):

(5) $${µ}_{d}=\mu_{m}\left(\frac{1-v_{f}}{1+1.5v_{f}}\right),$$

where:

νf is the volume fraction of dispersed-phase particles (aggregate).

According to Stokes’ law [45], the uniformity of dispersed systems can be expressed through the segregation rate, or particle sedimentation velocity; see Equation (6):

(6) $${Ʋ}_{s}=\frac{1}{18}\frac{D_{f}^{2}g(\rho_{f}-\rho_{m})}{\mu_{m}},$$

where:

ρm and μm are the density and viscosity of the mortar paste.

The dependence of μd, characterizing the workability of mortar containing hollow aggregates, on W/(L+C) and νf is shown in Figure 8. The effect of hollow aggregate size and W/(L+C) on υs, which characterizes the rate of loss of mortar uniformity, is also described graphically in Figure 8.

The viscosity values for mortar containing hollow filler (Figure 8) show that W/(L+C) has a greater influence than hollow aggregate volume fraction. Viscosity decreased consistently as W/(L+C) increased, and increasing hollow aggregate content also reduced viscosity. The segregation analysis (Figure 9) showed that, as lime–cement suspension viscosity varied (Table 15), the hollow aggregate exhibited equilibrium or segregation rates of up to (15–925) × 10^−7^ m/s at the maximum W/(L+C).

Depending on the required dry-mix density, dense aggregate components, particularly quartz sand, were also included. Like hollow aggregate particles, dense particles tend to sediment in the lime–cement suspension. Table 16 gives the calculated velocities of dense fine aggregate particles and shows the dependence of segregation intensity on particle size.

Table 16 shows that increasing W/(L+C) from 0.10 to 0.30 increases the settling velocity of quartz sand particles in a cement suspension with a viscosity μm = 2.1–10.2 Pa·s by almost fivefold. The rise in large hollow aggregate particles in the lime–cement suspension (υs = 925 × 10^−7^ m/s) should also be considered; limiting quartz sand particle size is therefore recommended to improve uniformity. When both dense and hollow aggregates fill the lime–cement suspension, segregation results from particles of different densities moving in opposite directions [55]. The test error was within 3%.

To justify binder selection, a thermal-insulating dry-mix structure was considered in which binder particles have equal sizes, microspheres have equal sizes, and adjacent microspheres are separated by a binder layer of constant thickness. The intermicrosphere binder layer is then two binder-particle diameters thick, while each microsphere is coated by a layer one binder-particle diameter thick. This composite structure forms because the high pozzolanic activity of microspheres causes binder particles to migrate toward their walls during initial structure formation. Hollow glass microspheres (HGM) were investigated as fillers in shell limestone-containing thermal-insulating dry mixes. On this basis, the optimum microsphere content was calculated and the density of the resulting composite was predicted (Table 17).

The calculated values were compared with experimental results: at an HGM content of 25% of the lime mass, composite density was 700 kg/m^3^; at 30% HGM, it was 840 kg/m^3^. The difference between calculated and measured densities is explained by the simplifying assumptions used in the calculations.

The processing and service properties of thermal-insulating dry-mix coatings are largely determined by their pore structure, which is strongly influenced by the water demand, nature, and structure of the filler.

Changes in the water demand of lime-based compositions with increasing microsphere content were investigated. Formulations with equal plasticity and different filler contents were prepared. Yield shear stress was measured using a KP-3 cone plastometer (Figure 10).

Figure 11 compares the volumes of open and closed pores in composites containing different fillers. All fillers were used at 40% of the lime mass.

Figure 11 shows that formulations containing hollow glass microspheres (HGM) and combined glass and aluminosilicate microspheres (HM) have lower water demand than those containing conventional sand. This is explained by the small proportion of open micropores that actively absorb water and the high volumetric packing efficiency provided by the microsphere shape. Closed pores arising from the hollow fillers predominate in microsphere-containing formulations. To assess the crack resistance of coatings with different fillers, shrinkage deformation (Figure 12), ultimate extensibility, and cohesive strength (Figure 13) were determined. Microsphere fillers improved crack resistance by reducing shrinkage during hardening and increasing ultimate extensibility and cohesive strength.

Sika-3180 superplasticizer was included in the complex modifier (CM) to improve rheological and processing properties. White cement and Zincum-5 hydrophobic powder were added to improve coating water resistance. Neolit P4400 redispersible powder improved cohesive and adhesive properties and the strength of the developed coatings.

Microsphere-containing coatings showed greater water resistance, lower moisture diffusion coefficients and capillary water absorption, and lower vapor permeability than composites with conventional sand aggregate. This was attributed to the predominance of closed porosity in the microsphere-containing composites.

The effects of fillers on heat-transfer mechanisms and moisture-transport kinetics were investigated. The emissivity ε, which characterizes the ability of the coating to exchange radiative heat, was measured using a Testo 882 thermal imager (Titisee-Neustadt, Germany) and a TEN-5 electronic thermometer (Moscow, Russia). Filler structure did not affect the measured emissivity, which was ε = 0.9.

Moisture transport kinetics through the mortar layer were established by examining changes in differential moisture content during wetting. A coating specimen consisting of four parallel disks, each 5 mm thick, was placed between two chambers. Chamber 1 was maintained at t_1_ = 60 °C and relative humidity φ_1_ = 100%; chamber 2 was at t_2_ = 20 °C and φ_2_ = 50%.

Moisture was more uniformly distributed through the HM-based coating than through the sand-based coating, S (Figure 14).

Thus, the HM-based coating better resists temperature- and moisture-induced deformation within the coating layer.

## 4. Discussion

Shell limestone waste was crushed to fractions of 0.63–5.0 mm and added to the lime–cement mixture. To quantify the mechanical properties of the thermal-insulating lime–shell limestone–cement composition (TLSCC), crushed shell limestone content P, as a percentage of mixture mass, was varied as follows: 2, 5, 7, 10, 12, 15, 17, and 20%. Mechanical test results are shown in Figure 15, Figure 16 and Figure 17, and regression equations describing density, flexural strength, and compressive strength are given in Table 18.

The results show that the shell limestone and hollow glass microsphere composite has low thermal conductivity and is suitable for use in thermal-insulating dry-mix mortars.

Table 19 and Table 20 present the optimum thermal-insulating dry-mix formulations and the main quality indicators of the resulting mortars.

Control specimens were also prepared for all the above formulations without adding Portland cement at 4% of the lime mass. All specimens hardened under air-dry conditions at 18–20 °C and 60–70% relative humidity.

The strength development of lime–cement specimens containing CM is shown in Figure 18.

The curves in Figure 18 show that all developed formulations had increased compressive strengths, ranging from 4.06 ± 0.137 to 3.75 ± 0.126 MPa. Formulation No. 1 had the highest strength, attributed to the optimum binder content of approximately 20% lime and an additional 4% white Portland cement, together with an effective proportion of 40% finely dispersed fillers and 16% CM.

Finely dispersed fillers, hollow glass microspheres, and CM, at the selected proportions of mixture mass, positively affected the principal properties of the thermal-insulating mortar and confirmed their effectiveness.

The effect of CM on the rheological properties of the shell limestone- and hollow microsphere-based thermal-insulating composite was investigated (Figure 19). The formulations behaved as plastic systems described by the Shvedov–Bingham equation, Equation (7):

(7) τ = τ_0_ + ηγ,

where:

τ_0_ is the yield shear stress, Pa;

η is the plastic viscosity, Pa·s;

γ is the shear rate, s^−1^.

Incorporating shell limestone, hollow microspheres, and CM at suitable contents accelerated the development of plastic strength (Figure 19).

Table 21 presents experimental dynamic shear stress data for the thermal-insulating lime–shell limestone–cement material as a function of shell limestone and CM contents.

Table 21 shows that shell limestone and hollow microspheres combined with CM have a structuring effect during hardening. Shear stress and plastic viscosity increased in their presence. At 200–400 rpm, the control without shell limestone, hollow microspheres, and CM had a dynamic shear stress of τ = 0.000187 MPa, compared with τ = 0.000312–0.000323 MPa for Formulas (1)–(3). This confirms their combined positive effect on hardening and structure formation.

Thermal conductivity results obtained using the ITP-MG4 instrument are presented in Table 22.

The data in Table 22 confirm that the developed shell limestone- and hollow glass microsphere-based mortars have lower thermal conductivity than the control and are suitable for thermal insulation applications.

One of the main properties of thermal-insulating dry-mix coatings is adhesion to the finished surface. Adhesion strength is determined by normal pull-off or shear testing. Service life and protective performance also depend substantially on adhesion strength and its stability.

During hardening and structure formation, the chemical and phase composition, structure, and properties of the system change. Macromolecules undergo conformational changes, the substrate surface becomes activated, and stresses develop in the contact zone. These factors influence adhesive interaction to varying degrees. Bonds formed initially may be retained, broken, or supplemented by new bonds during adhesion development and service. Adhesion depends not only on the presence but also on the number of bonds between contacting bodies, which is determined by the actual contact area between the mortar and substrate.

The bond strength of the developed lime–shell limestone–cement thermal-insulating mortars to concrete and cement–sand substrates was investigated (Table 23).

The developed formulations outperformed the control. Adhesion strength ranged from 0.32 to 0.37 MPa on concrete and from 0.34 to 0.40 MPa on cement–sand substrates. These values were achieved through shell limestone, hollow microspheres, and CM, which contribute to material service performance.

CM and the additives altered bonding capacity and improved cohesive–adhesive interactions with different substrates. Finely dispersed fillers also had a positive effect. Active centers formed from the chemical interaction of nanoscale diatomite silica particles with Ca(OH)_2_ and their adsorption at phase interfaces inhibited lime crystal growth and recrystallization. Diatomite and shell limestone powders promoted the principal hardening products: hydrate phases chemically similar to C–S–H(II) and calcite. Shell limestone and hollow microspheres, which possess pozzolanic activity, provided an additional contribution.

To assess service resistance, formulation No. 1 was subjected to repeated freezing and thawing after 28 days of air-dry hardening at (23 ± 5) °C and (65 ± 5)% relative humidity. Specimens measuring 70.7 × 70.7 × 70.7 mm were tested in series. Under the basic method, the required frost resistance grade was considered achieved when mean compressive strength after the specified number of freeze–thaw cycles decreased by no more than 10% relative to the control (Table 24).

The lime–shell limestone–cement thermal-insulating coatings withstood 35 cycles. After 35 freeze–thaw cycles, cube specimens showed a 9.2% loss in compressive strength, corresponding to grade F35 because the allowable loss was no more than 10%. The test error was within 2%.

Contact-zone frost resistance is a principal durability characteristic of finishing mortars. Composite specimens applied to concrete and cement–sand substrates were repeatedly frozen and thawed. Coating adhesion was assessed by shear and dolly pull-off tests. Results for formulation No. 1 are presented in Table 25.

Table 25 indicates a contact-zone frost resistance grade of F35 because strength loss did not exceed 20%; adhesion strength decreased sharply at 50 and 75 cycles.

During formulation development, the effects of the water-to-lime ratio W/L, modifying additive contents, and production technology on thermal-insulating properties were assessed. A thermodynamic analysis of possible reactions among lime, finely dispersed fillers, and mixing water was performed according to the second law of thermodynamics. Calculations confirmed the occurrence of filler–lime reactions. Qualitative X-ray diffraction analysis using a D8 ADVANCE diffractometer (Karlsruhe, Germany) was performed to investigate solid-phase reactions during structure formation (Figure 20).

Analysis of the diffraction peaks identified lines (Å) of calcium silicate hydrates C–S–H(I) and C–S–H(II), portlandite, calcite, and hydrohalite in hardened lime with finely dispersed fillers. The diffraction pattern is interpreted in Table 26.

The diffraction pattern confirms chemical interaction between the fillers and lime. After 28 days of air-dry hardening, free lime content was 63% in the control specimens and 28% in specimens with a lime-to-filler ratio of 1:0.4 and W/L = 0.9.

Differential thermal analysis (DTA) provided additional confirmation. Between 400 and 700 °C, the filler-containing hardened lime specimens showed calcium hydroxide decomposition and calcite dissociation, together with dehydration of C–S–H(I) and C–S–H(II) at 454.2, 471.2, 541.2, and 645.7 °C. The resulting products were calcium silicates and their modifications (Figure 21).

Calcium carbonate decomposition is a well-known process. It occurs in a single stage with carbon dioxide release:

CaCO_3_ → CaO + CO_2_↑

For Ca(OH)_2_, a deep endothermic peak indicates an energy requirement of 65.3 kJ/mol for decomposition. Water removal is accompanied by sample mass loss. The Ca(OH)_2_ mass-loss curve has two stages. The first accounts for 24% of the sample mass and corresponds to water release during calcium hydroxide decomposition between 425 and 560 °C. The second, approximately 2%, corresponds to calcium carbonate decomposition; this impurity is present in many calcium compounds because CO_2_ is continuously present in air. DTA and mass-loss curves are presented for CaO_2_ prepared according to the formulation. Its DTA curve shows a double endothermic peak beginning at 350 °C, with minima at 405 and 490 °C. Corresponding changes are observed on the CaO_2_ mass-loss curve.

Mass loss in the filler-containing hardened lime specimens within this temperature range was 25%. The substantial water-related mass loss supports the formation of numerous low- and high-basicity calcium silicate hydrates through chemical reactions between the fillers and lime.

The use of hollow glass microspheres in construction materials may, during service and under certain exposure conditions, potentially contribute to alkali–silica reaction (ASR) over time and therefore warrants evaluation with respect to material performance.

The review by Xiao et al. [56] systematizes the use of waste-glass powder (GP) as a supplementary cementitious material in Portland-cement systems and as a precursor for alkali-activated materials. It reports an amorphous-silica content of approximately 70% in GP and considers its effects on mechanical properties, durability, reaction extent, and the thermodynamic modeling of hydration products. The review also notes that evidence on the durability of GP-containing alkali-activated materials remains insufficient, especially with respect to ASR-related expansion.

Dong et al. [57] compared the effects of recycled glass in different forms and particle-size ranges on the fresh, mechanical, microstructural, and durability properties of cementitious and geopolymer concretes. The review considers chemical attack, chloride transport, elevated temperature, freeze–thaw exposure, carbonation, efflorescence, abrasion, and ASR. The authors concluded that durability and ASR depend strongly on glass type, particle size, and replacement level. In contrast to coarse glass cullet, finely ground glass powder can improve durability through pozzolanic activity, matrix densification, and reduced porosity, thereby potentially mitigating ASR expansion.

Yang et al. [58] evaluated ASR in glass aggregate used specifically in dry-mixed concrete blocks and compared accelerated laboratory methods (ASTM C1260 and ASTM C227) with long-term field monitoring over 15 years. The morphology of ASR gel observed in laboratory tests was consistent with the long-term field observations. External gel was found in larger pores and internal gel in cracks within the glass aggregate; external gel could be accommodated in large pores without hazardous expansion.

Khan et al. [59] investigated ASR in waste-glass fine aggregate by using ASTM C1293 concrete-prism tests and compared alkali-activated systems with Portland-cement concrete. The one-year expansions of the OPC concrete and the ground-granulated-blast-furnace-slag alkali-activated concrete were 0.21% and 0.13%, respectively; both values exceeded the ASTM limit of 0.04%. In contrast, expansion in the fly ash–GGBFS system remained within the acceptable range. Calcium-rich ASR products were identified in the OPC and slag-based alkali-activated systems, whereas the FA–GGBFS system formed less deleterious gels with a high Al/Si ratio. These results show that ASR risk depends not simply on the presence of glass, but also on binder chemistry and the composition of reaction products.

The critical review by Hamada et al. [60] addresses the use of waste glass in conventional and high-performance concrete. It considers fresh-mixture properties, mechanical performance, and durability when glass is used as aggregate. The authors emphasize that performance depends on glass particle size and type, replacement level, and mixing and curing conditions. Glass powder was associated with improved durability through densification of the pore structure and microstructure. The review supports the use of recycled glass as fine or coarse aggregate but also demonstrates the need to optimize the formulation.

Although the studies cited above concern waste-glass powders and aggregates rather than hollow glass microspheres, they demonstrate the need to evaluate potential ASR risk in glass-containing cementitious systems. The risk is governed not only by the presence of amorphous silica but also by the chemical processes in the binder system and the composition of the reaction products.

No dedicated ASR tests were performed in the present study; therefore, ASR was not assessed or identified in the tested mortars. ASR evaluation, including its potential effects on the principal physical, mechanical, and thermophysical properties of hollow glass microsphere-containing thermal-insulating dry-mix mortars during service, is planned as a topic for future research.

## 5. Conclusions

The study yielded the following principal conclusions.

1. Mortar viscosity with hollow filler was strongly influenced by W/(L+C). Increasing hollow filler content and W/(L+C) reduced viscosity, with equilibrium or segregation rates of up to (15–925) × 10^−7^ m/s at the maximum W/(L+C). Formulations containing hollow glass microspheres had low water demand, which was attributed to the small proportion of water-absorbing open micropores and the high volumetric packing efficiency provided by the microsphere shape.
2. The ability of thermal-insulating coatings to resist radiative heat transfer was assessed through emissivity ε. Filler structure did not affect emissivity, which was ε = 0.9. The composite based on shell limestone and hollow glass microspheres had low thermal conductivity and is suitable for use in thermal-insulating dry-mix mortars.
3. The developed formulations had increased compressive strengths of 4.06 ± 0.137 to 3.75 ± 0.126 MPa. Formula (1) had the highest strength, attributed to approximately 20% lime plus 4% white Portland cement, 40% finely dispersed fillers, and 16% CM.
4. At 200–400 rpm, the control without shell limestone, hollow microspheres, or CM had a dynamic shear stress of τ = 0.000187 MPa, compared with τ = 0.000312–0.000323 MPa for Formulas (1)–(3). This demonstrates the combined positive effect of these components on hardening and structure formation. Adhesion strengths of 0.32–0.37 MPa on concrete and 0.34–0.40 MPa on cement–sand substrates were achieved through the incorporation of shell limestone, hollow microspheres, and CM, which contribute to service performance.
5. CM and the additives altered bonding capacity and improved cohesive–adhesive interaction with different substrates; finely dispersed fillers also contributed positively. Active centers formed by chemical interaction between nanoscale silica in diatomite powder and Ca(OH)_2_, together with adsorption at phase interfaces inhibiting lime crystal growth and recrystallization, promoted hydrate phases chemically similar to C–S–H(II) and calcite as the main hardening products. Shell limestone and hollow microspheres possess pozzolanic activity.

## Figures and Tables

**Figure 1:**
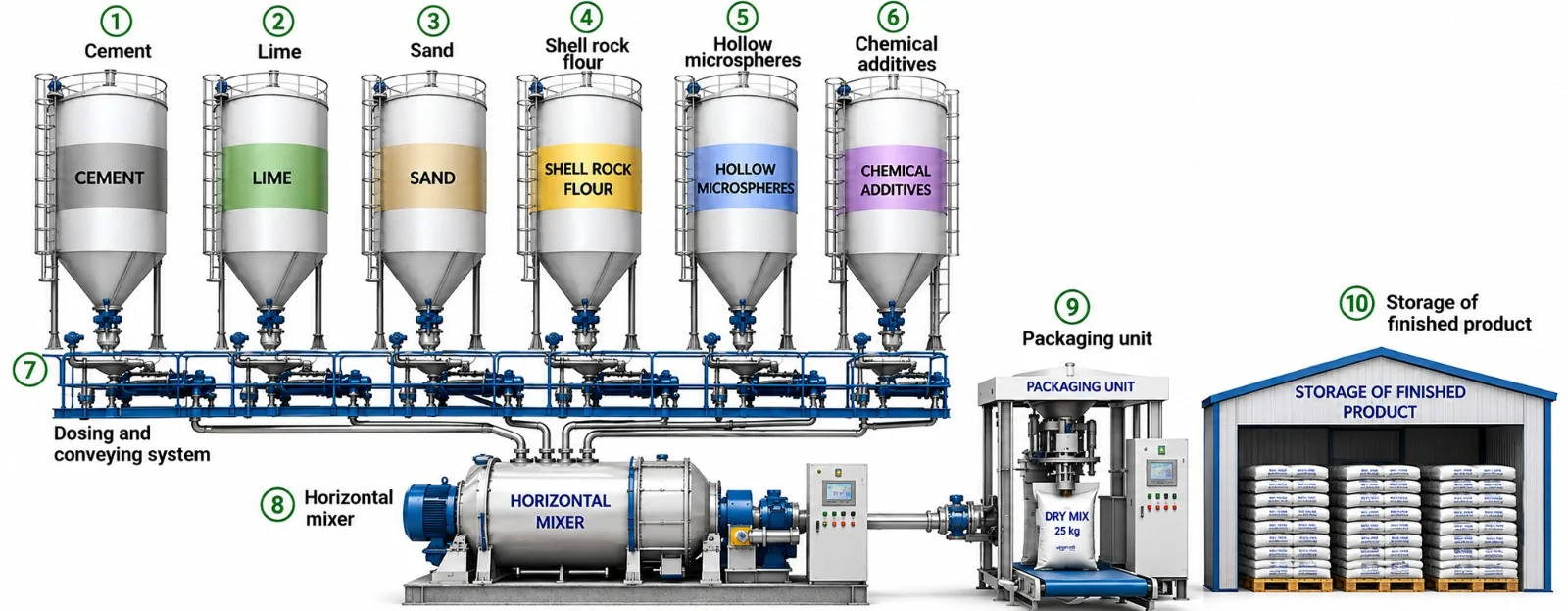
Process flow diagram for producing effective thermal-insulating dry-mix mortars. 1—Cement hopper; 2—lime hopper; 3—shell limestone hopper; 4—graded sand hopper; 5—polymer additive hopper; 6—complex modifier hopper; 7—rotor mixer; 8—packaging station; 9—finished product storage; 10—feeders.

**Figure 2:**
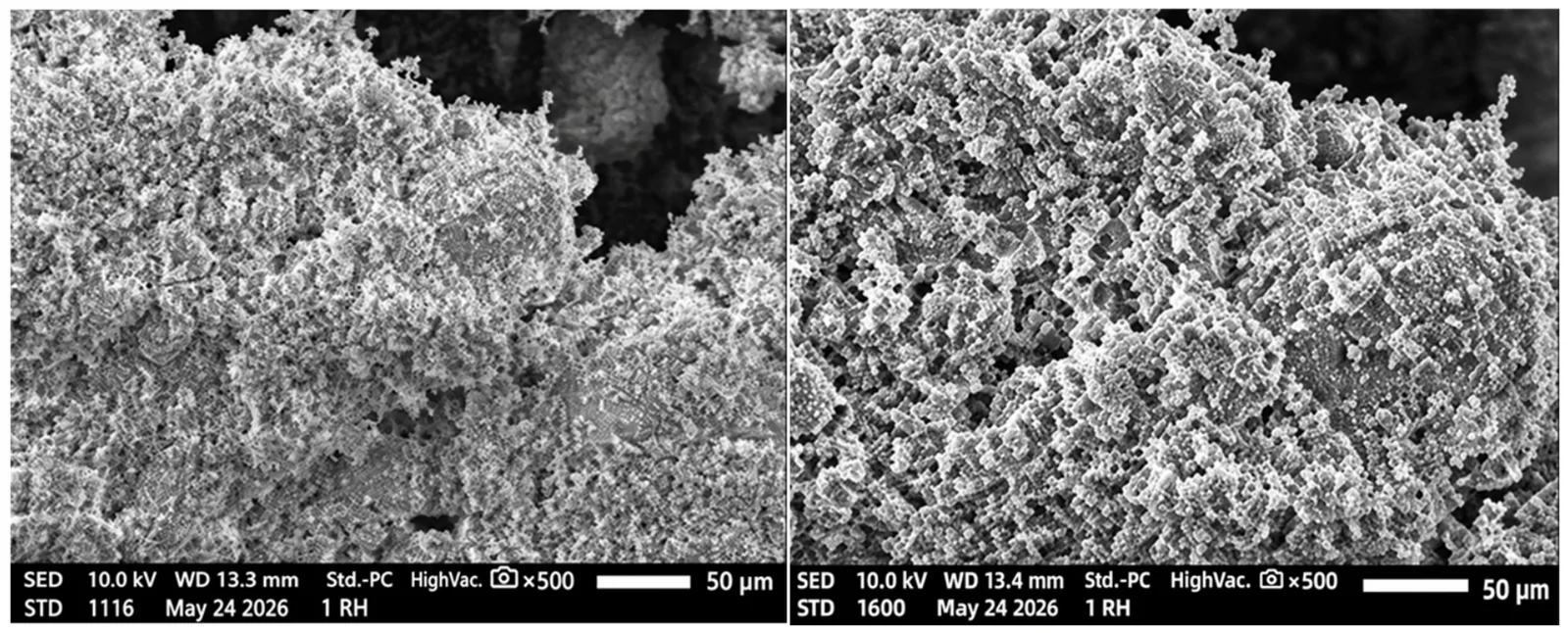
SEM images of shell limestone extraction waste from the Zhetybay deposit.

**Figure 3:**
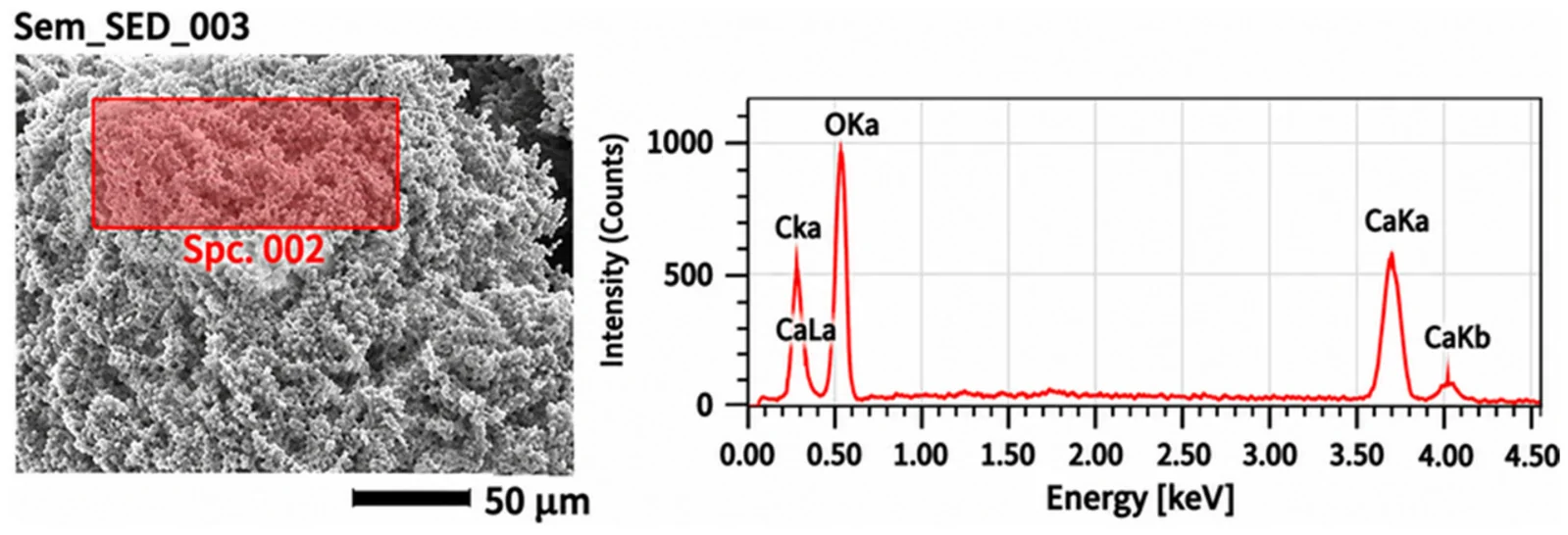
EDS spectrum of shell limestone extraction waste (area Spc_002).

**Figure 4:**
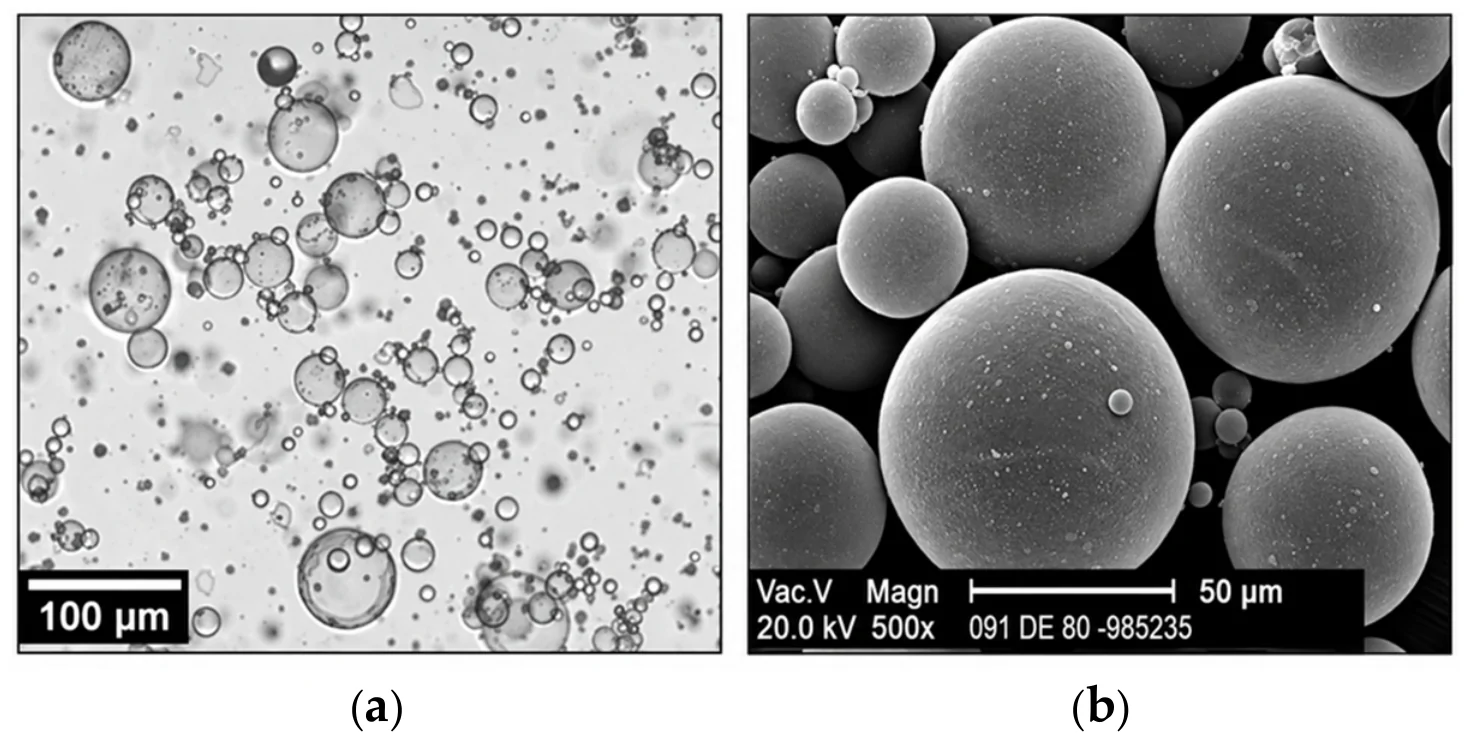
Electron micrograph of hollow glass microspheres [51]. (**a**) 20–100 µm. (**b**) 75 µm.

**Figure 5:**
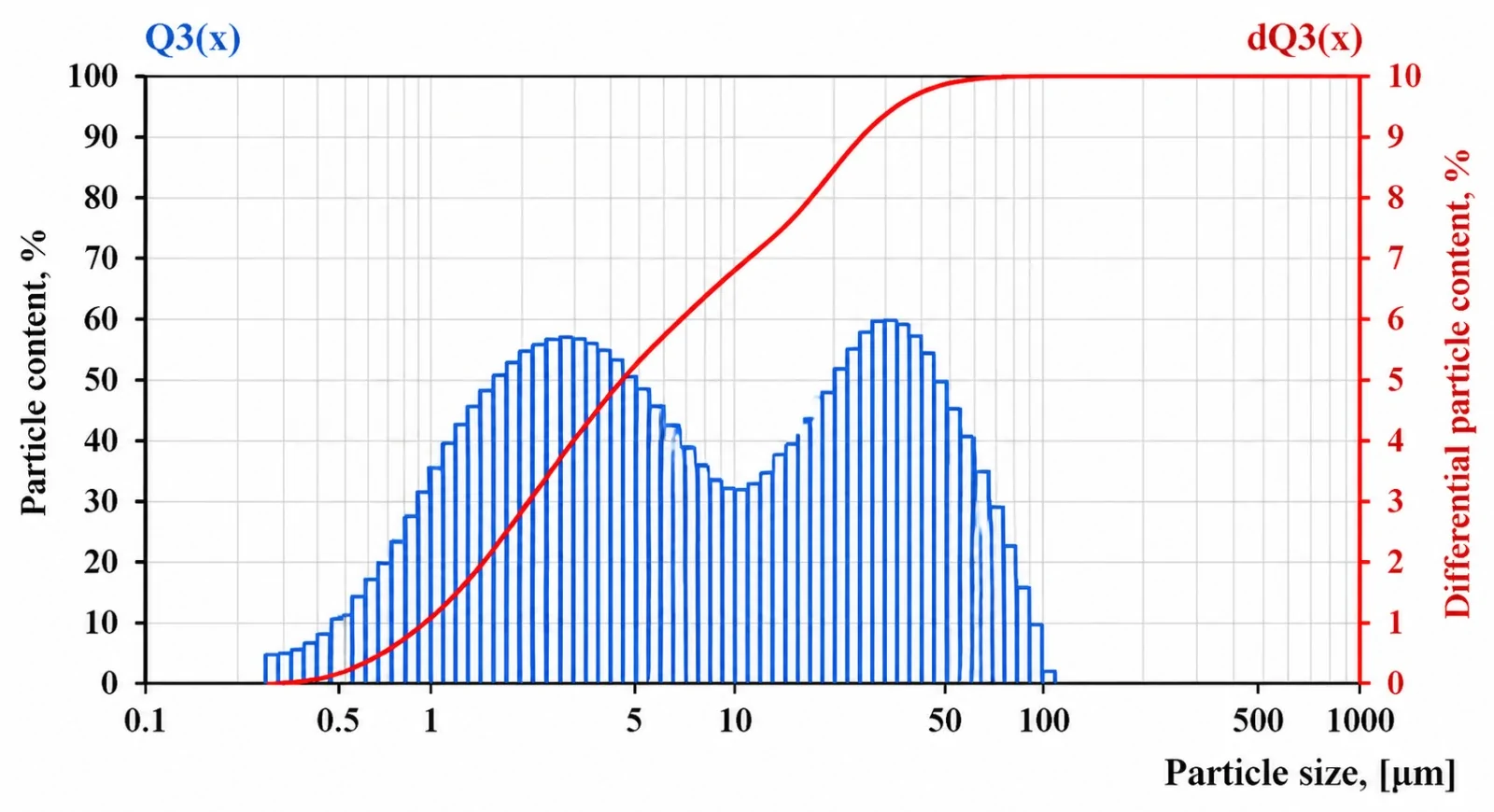
Particle-size distribution of shell limestone powder.

**Figure 6:**
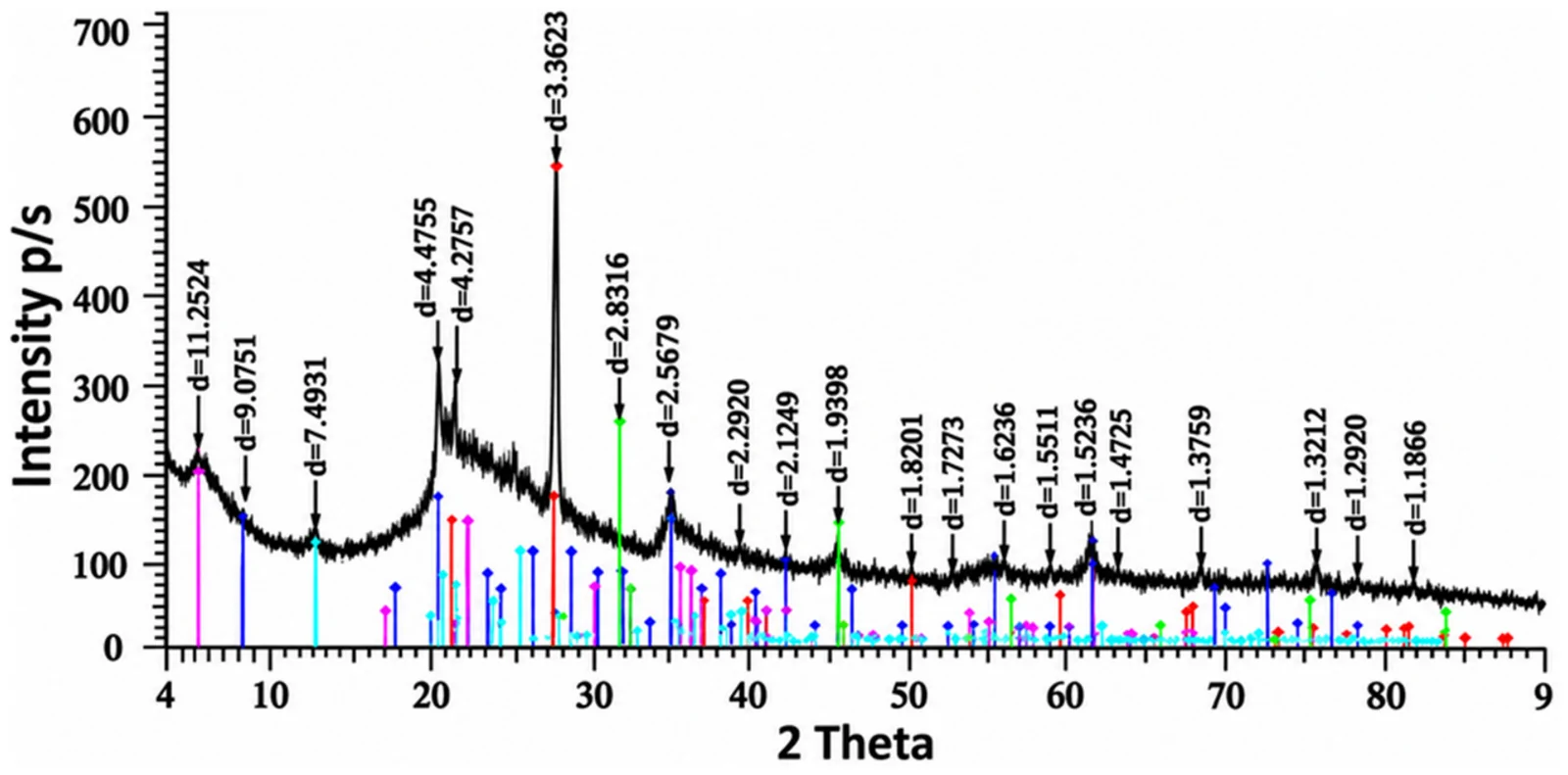
X-ray phase analysis of diatomite raw material from the Aktobe deposit [52].

**Figure 7:**
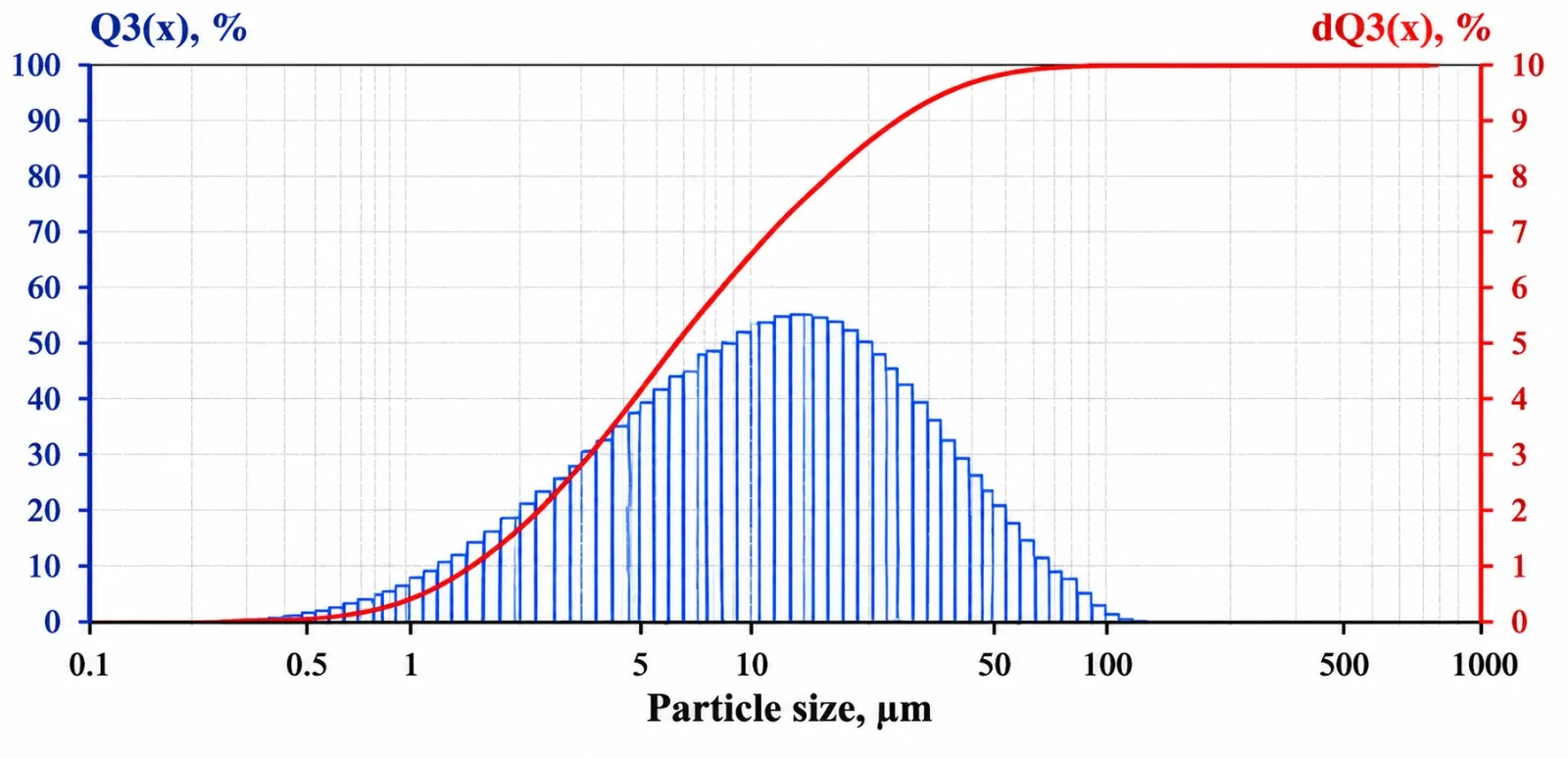
Particle-size distribution of diatomite powder.

**Figure 8:**
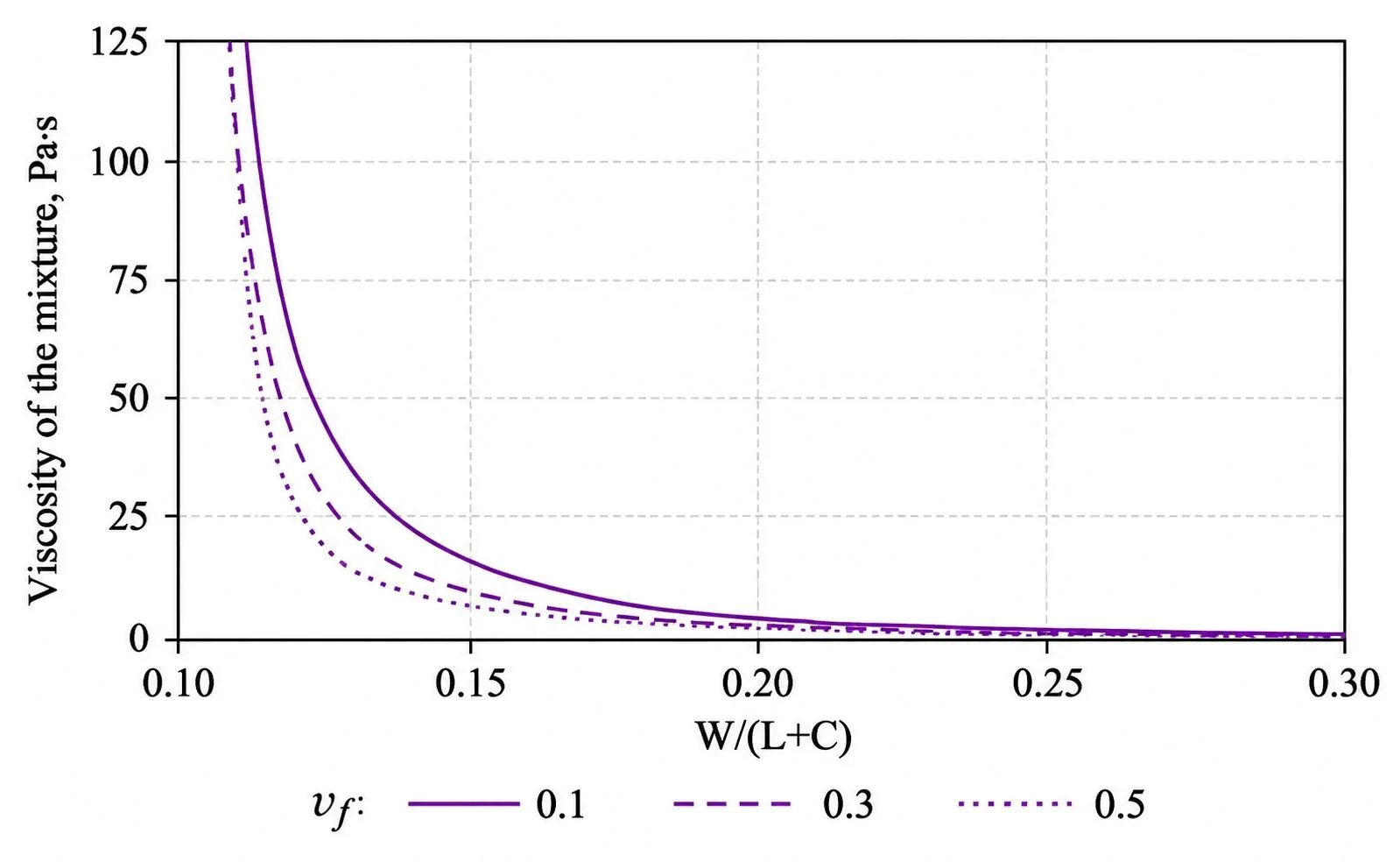
Dependence of mortar viscosity on W/(L+C) at different hollow filler contents.

**Figure 9:**
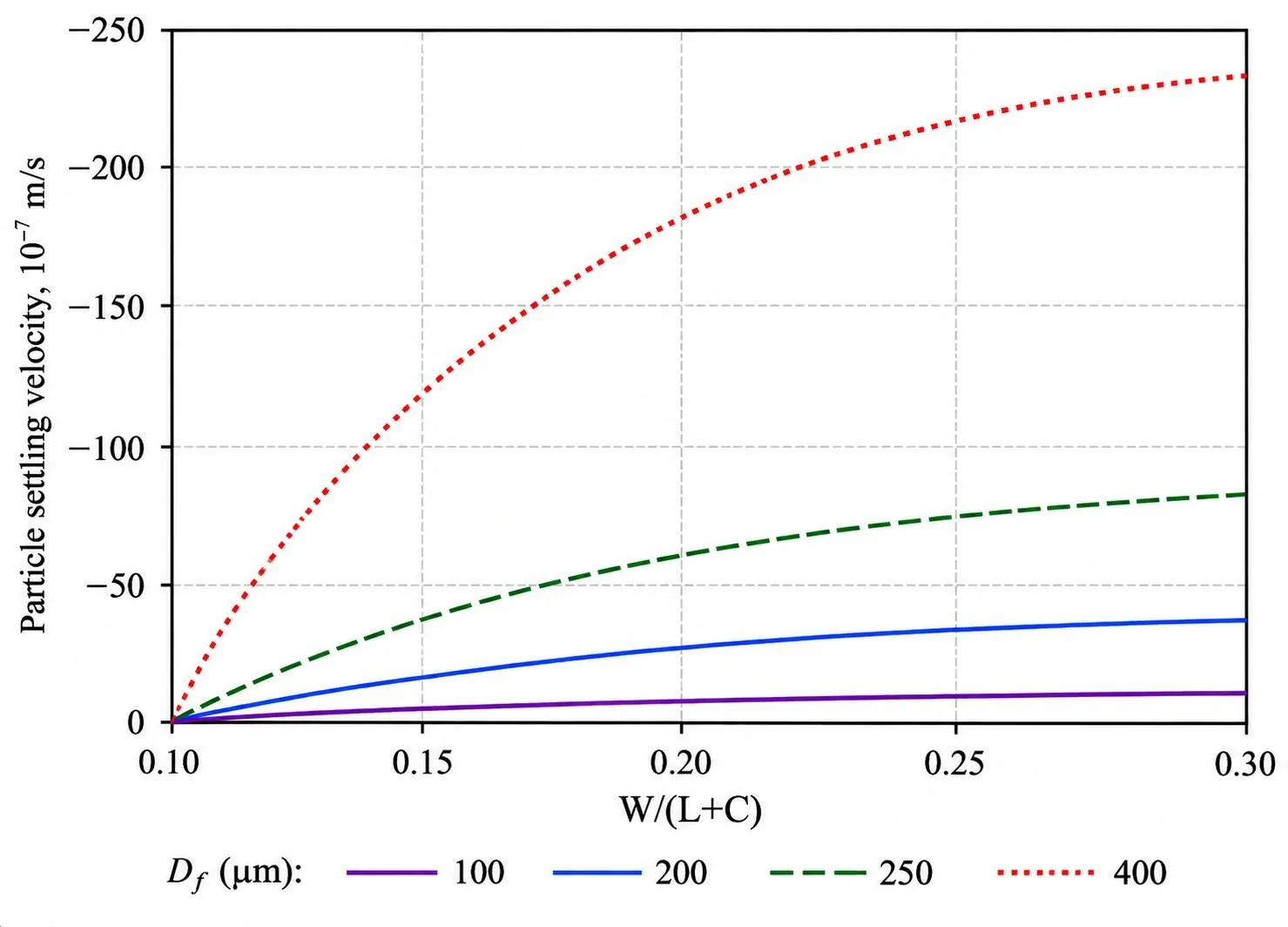
Sedimentation of hollow aggregate in the mortar suspension.

**Figure 10:**
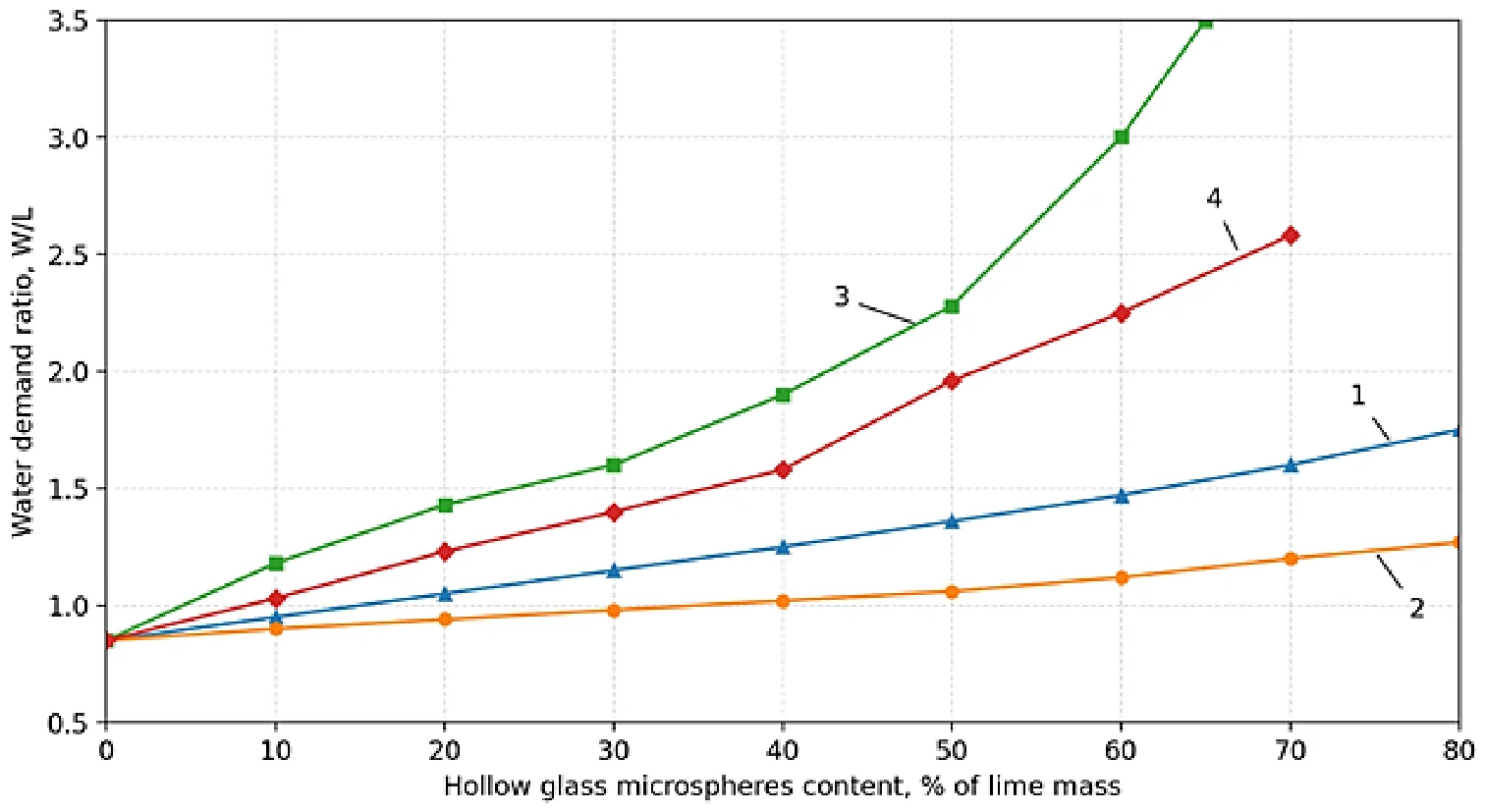
Water demand of lime–shell limestone compositions as a function of hollow microsphere content. 1—Hollow glass microspheres; 2—hollow aluminosilicate microspheres; 3—sand; 4—shell limestone + hollow microspheres.

**Figure 11:**
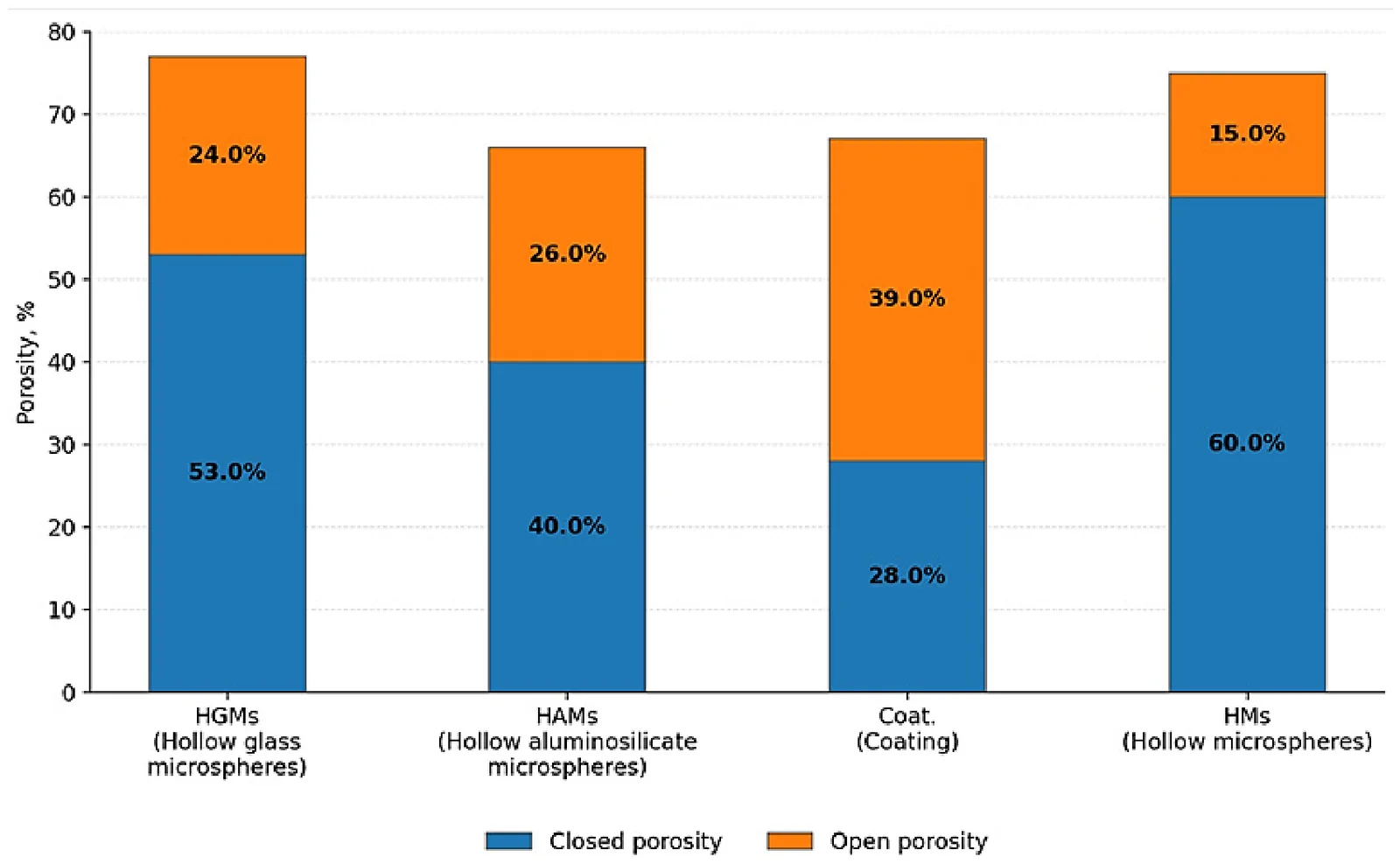
Pore volume ratios in lime–shell limestone–cement composites.

**Figure 12:**
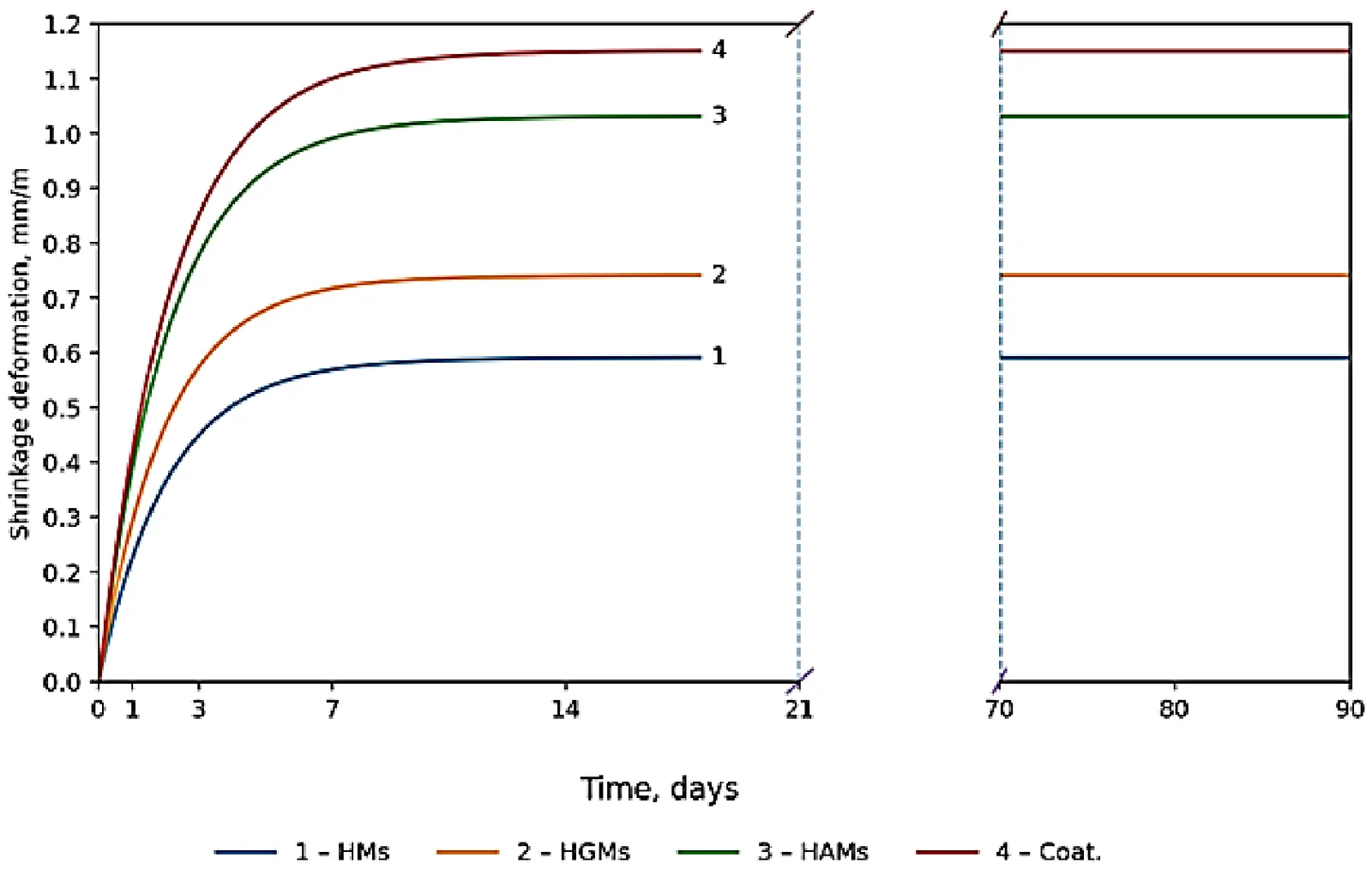
Shrinkage deformation kinetics of coatings with different fillers.

**Figure 13:**
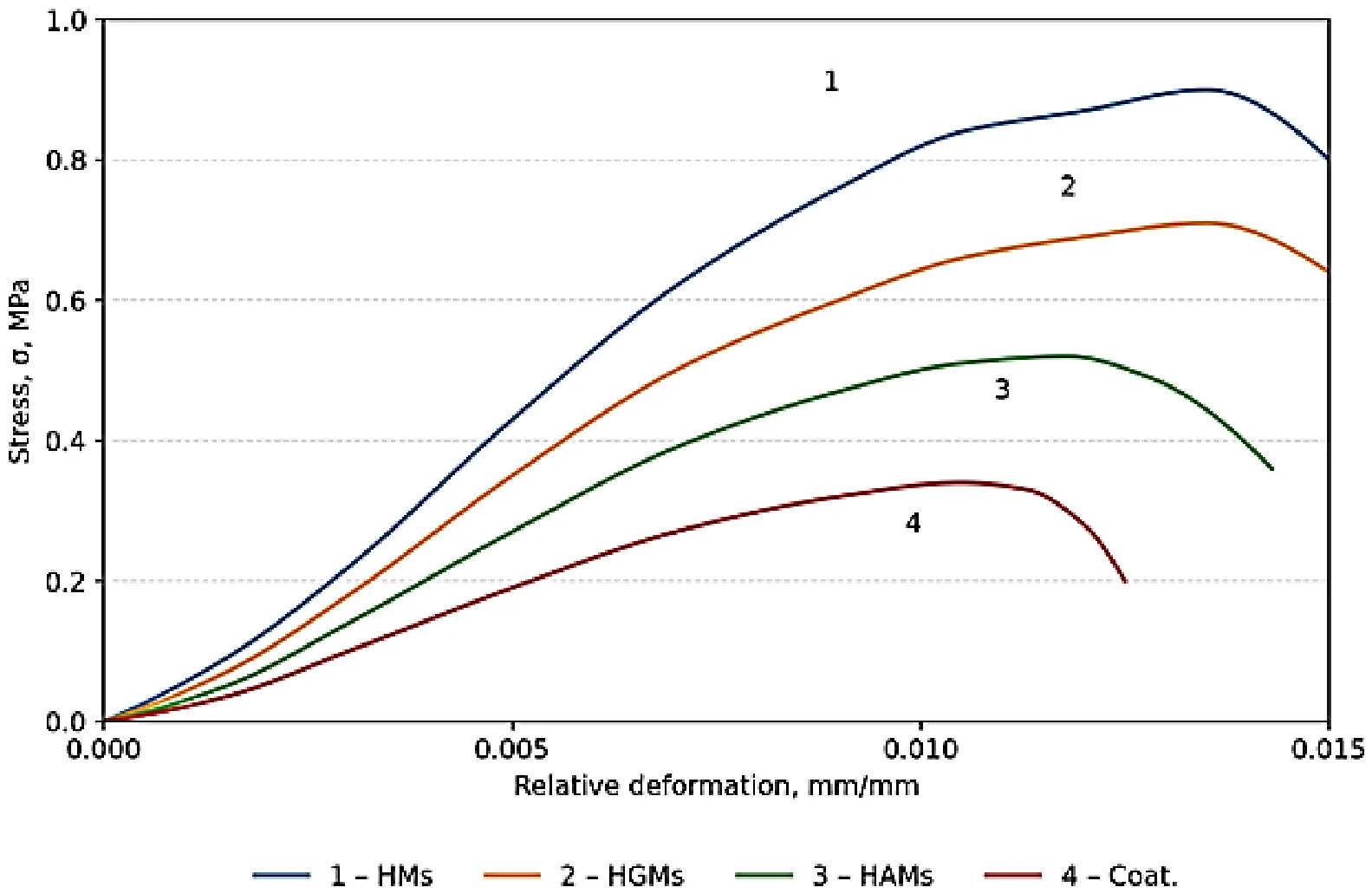
Evolution of relative tensile deformation in coating specimens with different fillers.

**Figure 14:**
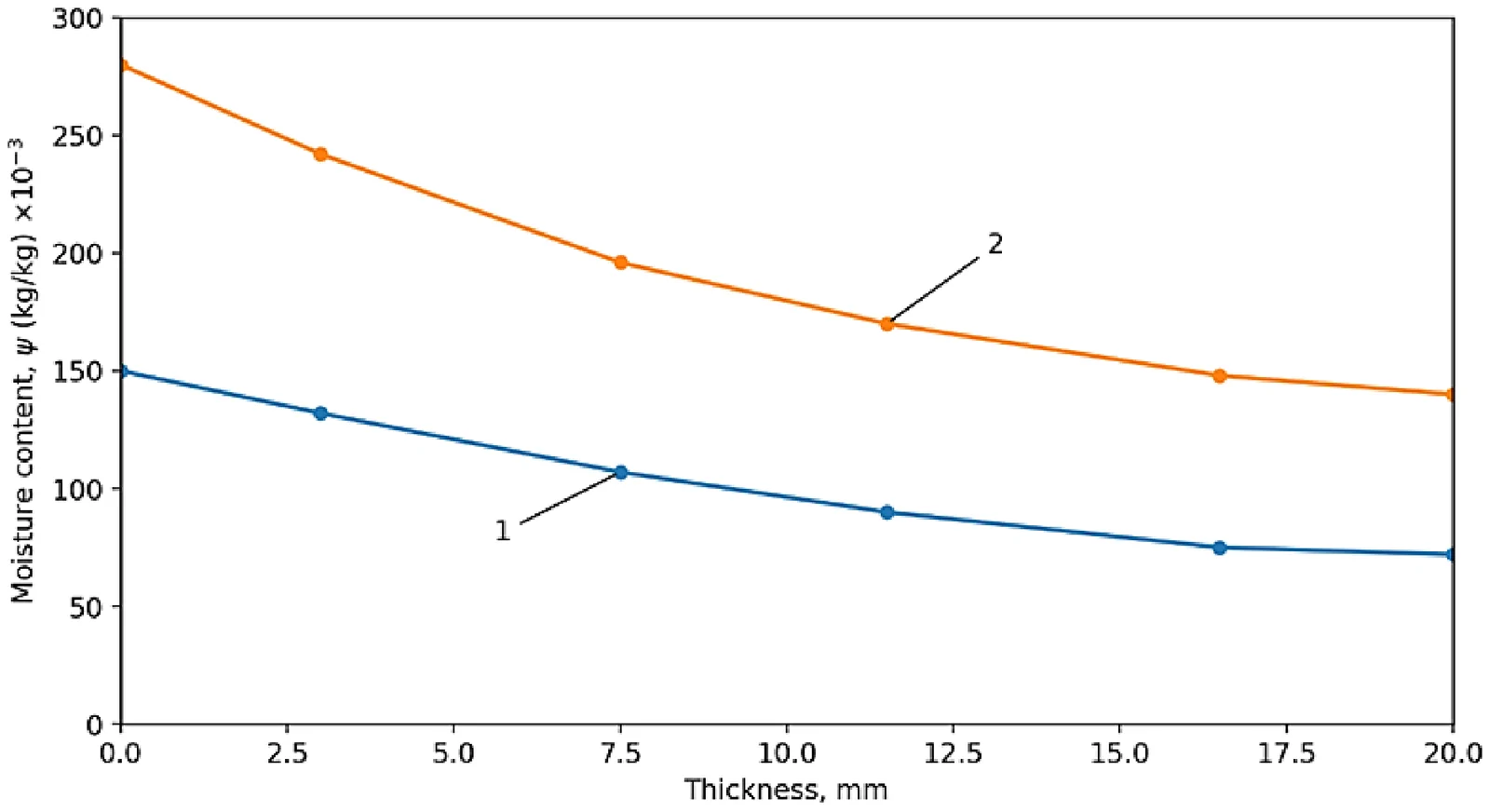
Moisture distribution through the thermal-insulating coating: 1—HM; 2—S.

**Figure 15:**
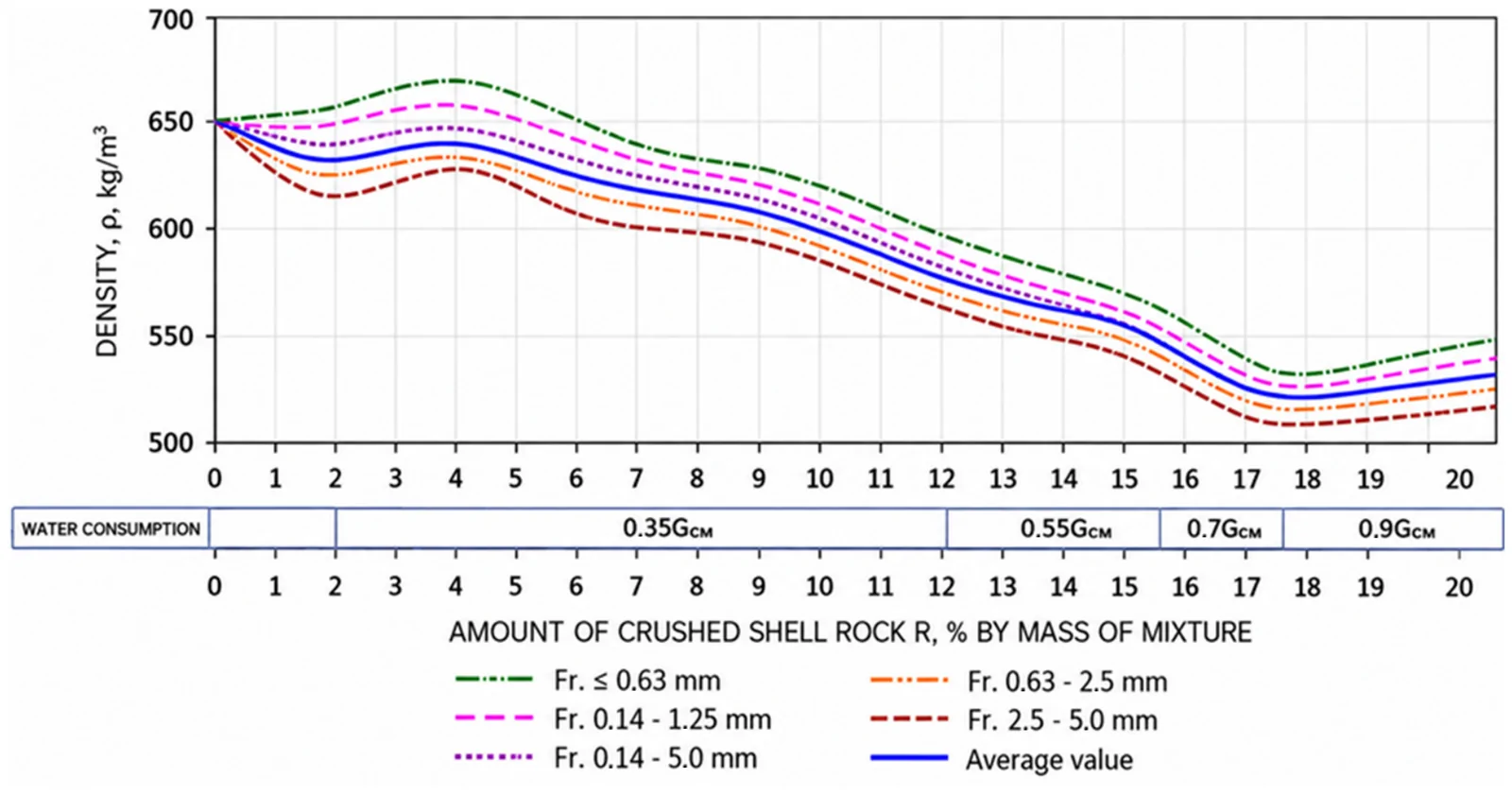
Density variation in the thermal-insulating lime–shell limestone–cement material with shell limestone content.

**Figure 16:**
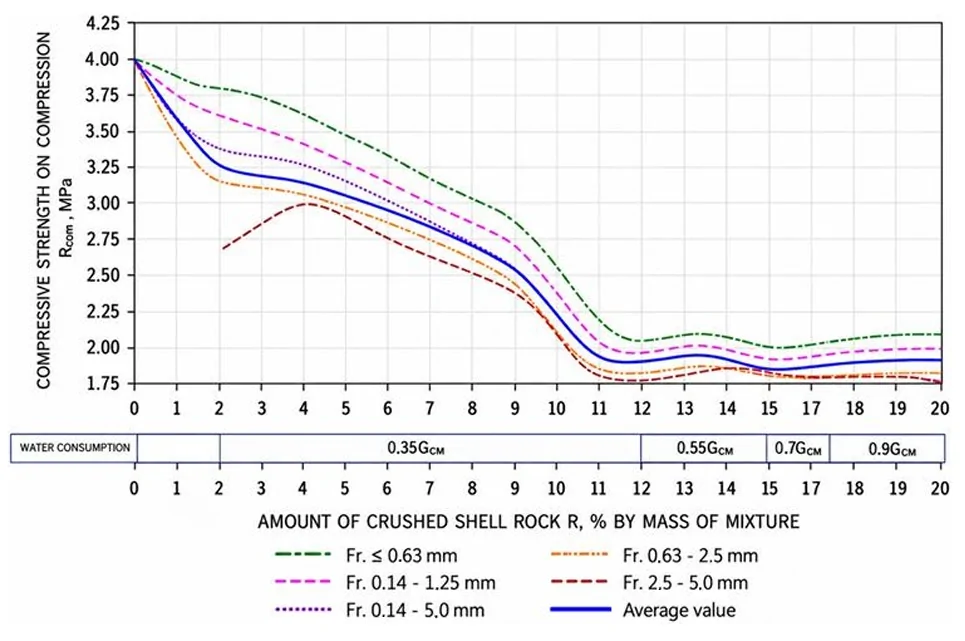
Variation in compressive strength of the thermal-insulating lime–shell limestone–cement material with shell limestone content.

**Figure 17:**
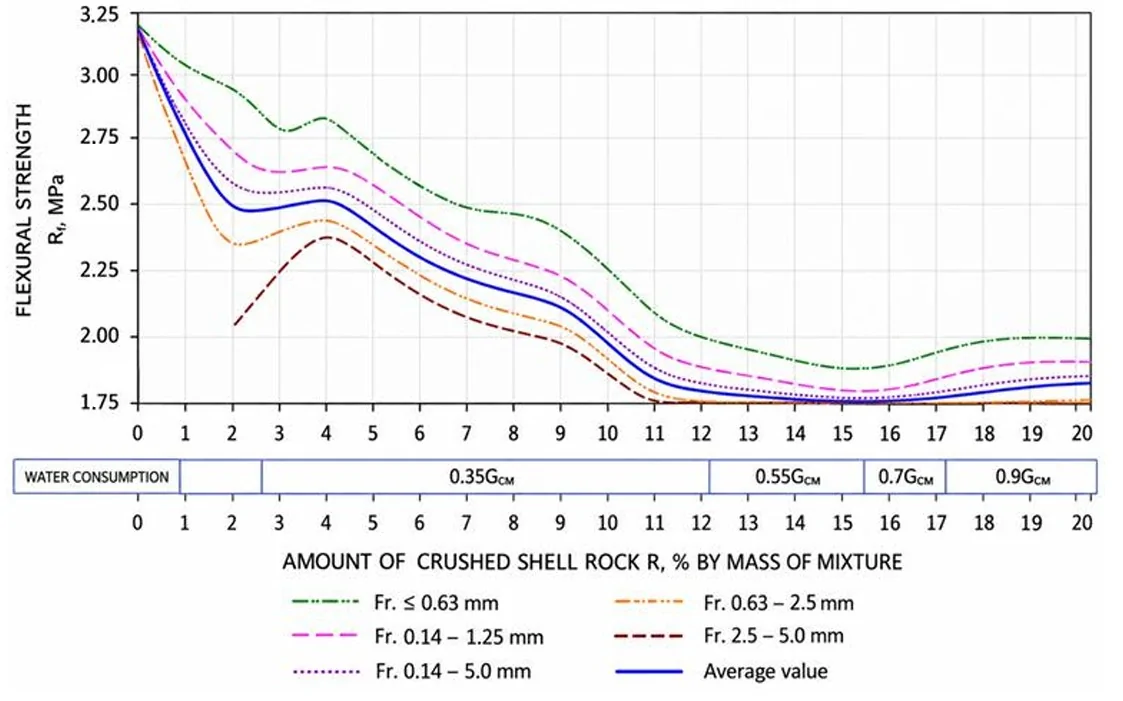
Variation in flexural strength of the thermal-insulating lime–shell limestone–cement material with shell limestone content.

**Figure 18:**
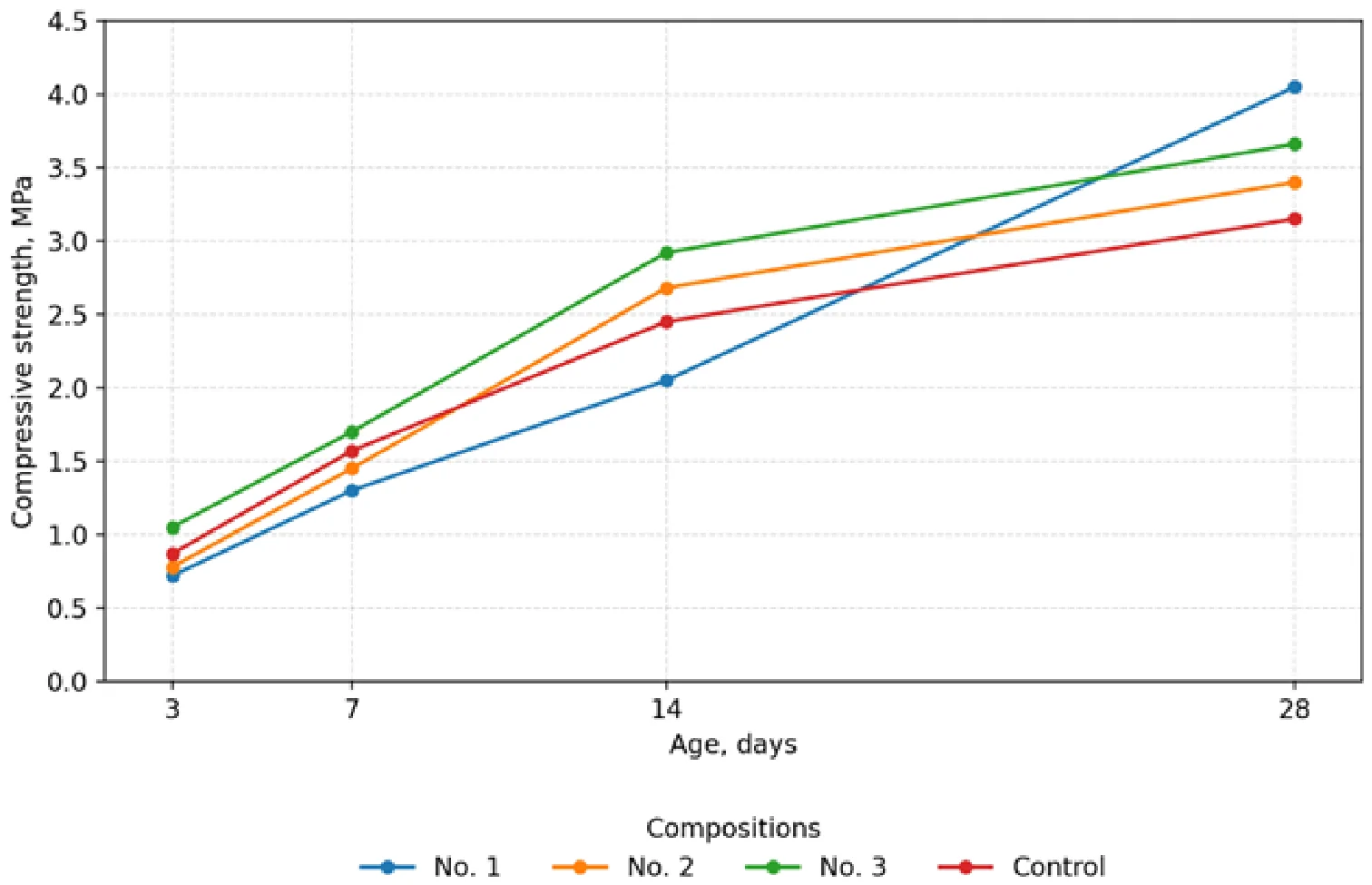
Compressive strength of thermal-insulating dry-mix compositions based on shell limestone, hollow microspheres, and CM.

**Figure 19:**
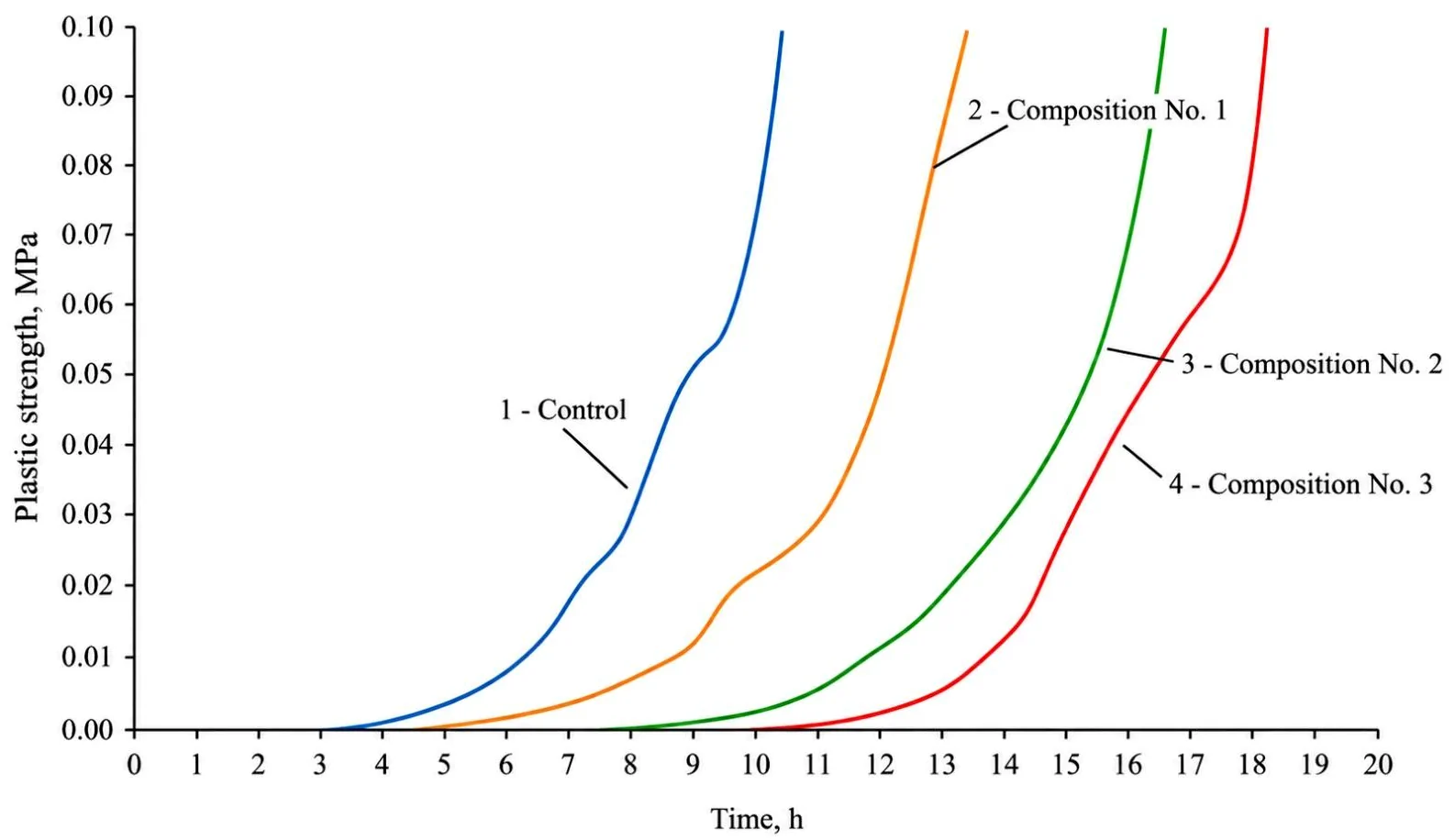
Plastic strength development of thermal-insulating dry-mix mortars with different formulations.

**Figure 20:**
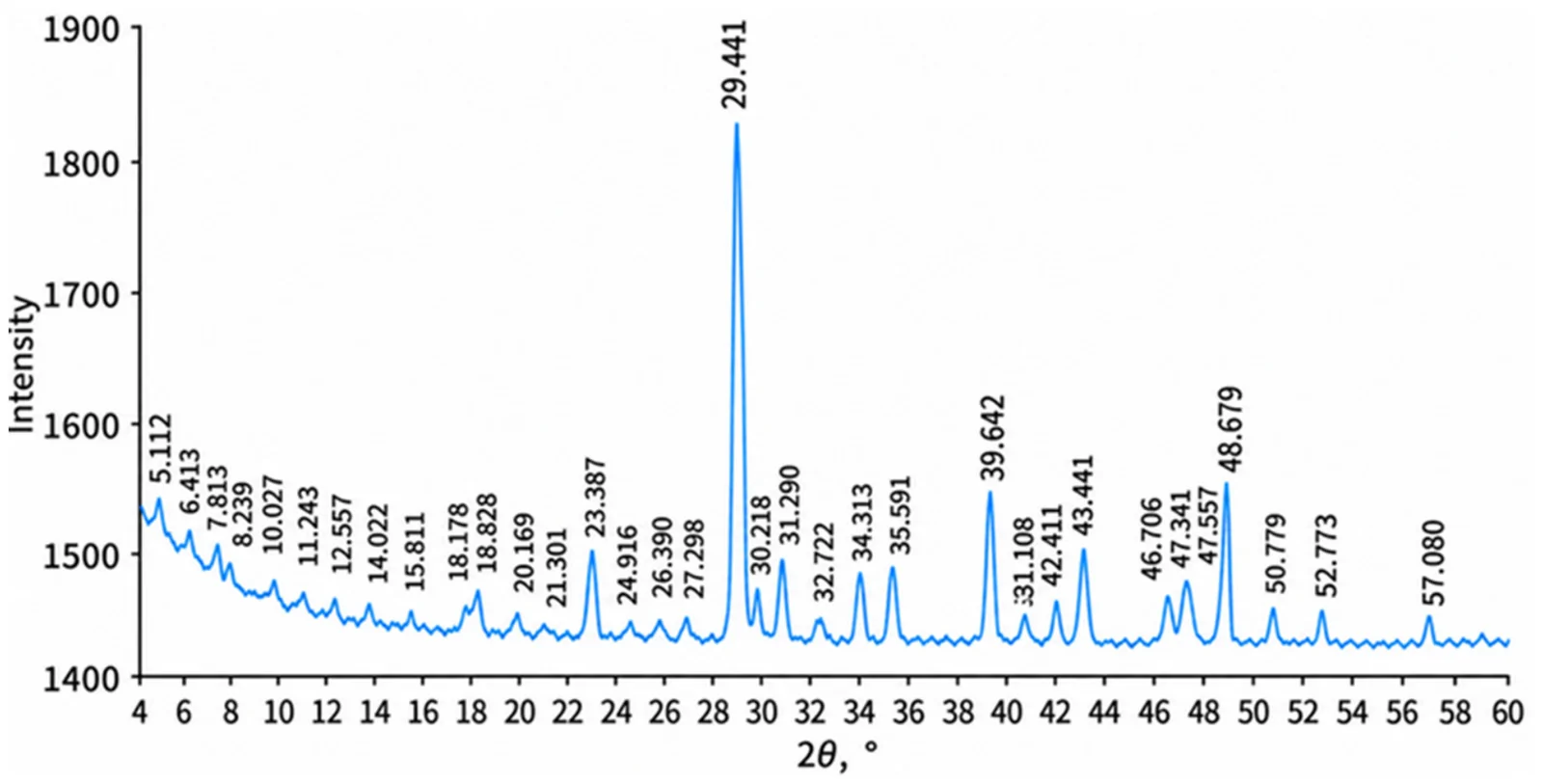
X-ray diffraction pattern of hardened lime containing finely dispersed fillers.

**Figure 21:**
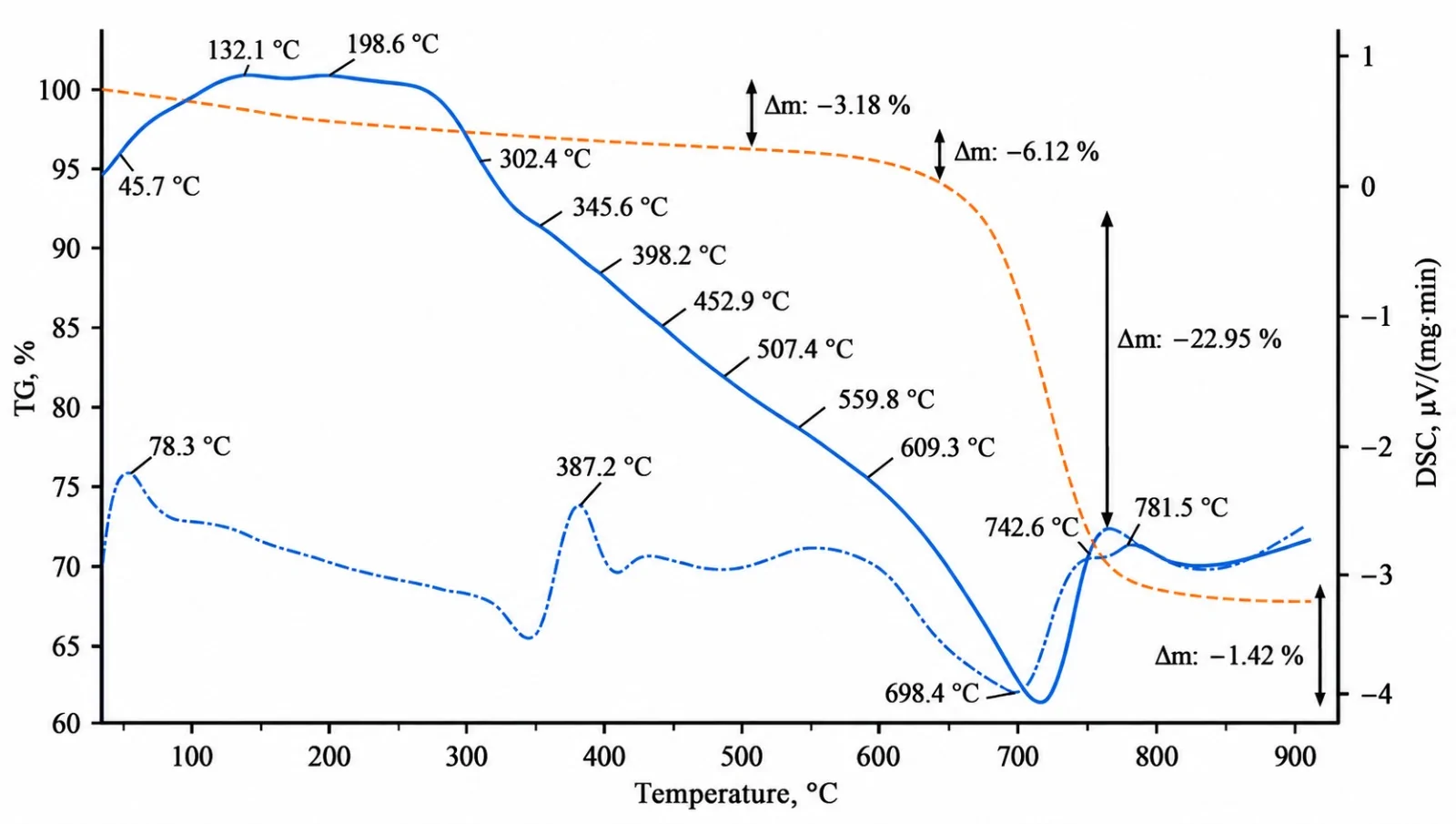
Thermogram of hardened lime with finely dispersed fillers.

**Table 10:** Chemical and mineralogical composition of diatomite.

Minerals	Quartz/SiO_2_	Halite/NaCl	Montmorillonite/Ca, Na	Muscovite/H_4_K_2_ (Al,Fe)_6_Si_6_O_24_	Kaolinite/Al_2_ (Si_2_O_5_)(OH)_4_
Content, %	42.9	17.9	≤1.0		

**Table 11:** Physicochemical properties of the mineral additive.

No.	Property	Value
1	Appearance	351
2	Activity, mg/g	689
3	Specific surface area, m^2^/kg	568
4	Bulk density, kg/m^3^	568.25
5	True density, kg/m^3^	2141

**Table 12:** Main properties of Sika-3180 superplasticizer.

No.	Property	Measured Values
1	Water reduction, %	40.4
2	Workability retention, min	180.5
3	Plasticizing effect without strength loss at any curing age, mm, minimum	125

**Table 13:** Main properties of Neolit P4400.

No.	Property	Neolit P4400 Values
1	Powder color	White
2	Solids content, %	98
3	Ash content, %	9
4	Stabilization system	Polyvinyl alcohol
5	Minimum film-forming temperature, °C	+6

**Table 14:** Main properties of Zincum-5.

No.	Property	Zincum-5 Values
1	Powder color	White to yellowish white
2	Zinc oxide content, %, minimum	13.8
3	Mass fraction retained on a 200-mesh sieve, %, maximum	0.2
4	Water content, wt.%, maximum	0.5
5	Density, g/cm^3^	266
6	Melting temperature, °C	121
7	Free fatty acid content, %, maximum	0.41
8	Saponification value, mg KOH/g	200

**Table 15:** Viscosity of the lime–cement suspension at different W/C ratios.

Property	Values				
W/(L+C)	0.10	0.15	0.20	0.25	0.30
μm, Pa·s	225	9.7	5.6	4.8	3.0

**Table 16:** Settling velocity of quartz sand particles in the lime–cement suspension.

W/(L+C)	υs at Different Df Values, ×10^−7^ m/s				
	0.16	0.315	0.63	1.25	2.5
0.10	1.9	5.1	43.3	96.4	382
0.20	4.8	20.1	121.1	679.3	1145
0.30	10.2	78.1	198.2	1119	1689

**Table 17:** Calculated properties of shell limestone-based thermal-insulating dry-mix mortars.

Binder	Filler	Microsphere Content, % of Binder Mass	Microsphere Volume in Dry Mix, Vm, %	Mixture Density, ρ, kg/m^3^
Lime	HGM	25	59.87	988.44
Cement	HGM	5	38.56	2375.7
Lime + cement	HGM	30	53.67	1448.6

**Table 18:** Physical and technical properties of the thermal-insulating lime–shell limestone–cement composition (TLSCC).

No.	Material Property	Property Regression Equations	Range of Component P	Correlation Coefficient
1	Mean density, kg/m^3^	γ_0_ = γ_0_p + 2.32·P^2^ − 11.6·P γ_0_ = 1147.56·e^−0.203·P^	0 ≤ P ≤ 2 2 ≤ P ≤ 20	0.958 0.976
2	Compressive strength, MPa	Rc = Rc,p·e^−0.203·P^	0 ≤ P ≤ 20	0.968
3	Flexural strength, MPa	Rf = Rf,p·0.0115·P^2^ − 0.0791·P Rf = 0.215·e^−0.113·P^	0 ≤ P ≤ 2 2 ≤ P ≤ 20	0.931 0.978

To obtain good moldability, the mixing-water-to-mixture ratio W/Gmix was selected according to P: for P = 0–12%, W/Gmix = 0.35; for P = 12–15%, W/Gmix = 0.700; for P = 15–17%, W/Gmix = 0.70; and for P = 17–20%, W/Gmix = 0.9.

**Table 19:** Optimum shell limestone-based thermal-insulating dry-mix mortar formulations.

Ingredient	Composition, %		
	No. 1	No. 2	No. 3
Lime	25	32.5	40
Cement	4	2.25	0.5
Shell limestone powder	15	17.5	20
Hollow glass microspheres	15	17.5	20
Sand	10	12.5	15
Neolit P4400 redispersible powder	5	7.5	10
Complex modifier CM	16	15.25	14.5

**Table 20:** Main quality indicators of mortars prepared from the thermal-insulating dry mix.

Property	Formulations			Control
	No. 1	No. 2	No. 3	
Mean density, kg/m^3^	600	580	560	670
Compressive strength, MPa	4.05	3.70	3.25	3.75
Adhesion to the substrate after air-dry curing, MPa, minimum	1.2	1.0	0.8	-
Flexural tensile strength, MPa, minimum	2.5	2.25	2.0	-
Water absorption coefficient, %, maximum	0.3	0.4	0.5	-
Frost resistance, cycles, minimum	50	50	50	-
Vapor permeability, mg/(m·year·Pa), minimum	0.04	0.045	0.05	-
Thermal conductivity, W/(m^2^ K)	0.113	0.1	0.096	0.140

**Table 21:** Dynamic shear stress of the thermal-insulating lime–shell limestone–cement material as a function of shell limestone and CM contents.

Formulations	Static Shear Stress, MPa	Dynamic Shear Stress, MPa, at Rotational Speed, rpm		Plastic Viscosity, Pa·s, at Rotational Speed, rpm	
		200–400	300–600	200–400	300–600
Control	0.00042	0.000187	0.000285	45.22	57.25
No. 1	0.00044	0.000218	0.000312	46.04	62.88
No. 2	0.00046	0.000220	0.000320	49.76	64.86
No. 3	0.00047	0.000225	0.000323	49.77	68.44

**Table 22:** Thermal conductivity of thermal-insulating dry-mix mortars.

Property	Formulations			
	Control	No. 1	No. 2	No. 3
Thermal conductivity, W/(m °C)	0.128	0.113	0.120	0.111

**Table 23:** Adhesive and cohesive properties of the thermal-insulating dry-mix mortar.

Formulation No.	Adhesion Strength, MPa		Cohesive Strength, MPa
	Substrate Type		
	Concrete Substrate	Cement–Sand Substrate	
Control	0.30	0.32	0.39
1	0.32	0.34	0.49
2	0.34	0.36	0.53
3	0.37	0.40	0.56

**Table 24:** Freeze–thaw cycles for the lime–shell limestone–cement thermal-insulating mortar.

Number of Cycles	Compressive Strength Loss, %	Frost Resistance Grade
5	3.0	-
10	3.4	F10
15	4.6	F15
25	5.3	F25
35	9.2	F35
50	11.0	F50
75	20.1	F75

**Table 25:** Contact-zone adhesion strength to the substrate after freeze–thaw cycles [52].

Number of Cycles	Coating Adhesion to the Substrate by Shear Testing	Coating Adhesion to the Substrate by Dolly Pull-Off Testing
0	0.303	0.354
5	0.300	0.350
10	0.298	0.340
15	0.296	0.329
25	0.296	0.310
35	0.295	0.322
50	0.229	0.275
75	0.085	0.174

**Table 26:** Interpretation of the X-ray diffraction pattern of hardened lime with finely dispersed fillers.

Diffraction Lines	Peak Values
(Å) Calcium silicate hydrates C–S–H(I) and C–S–H(II)	20.312; 14.853; 12.363; 10.983; 9.612; 8.672; 7.628; 6.511; 5.698; 5.324; 4.611; 3.746; 3.192; 3.048; 2.827; 2.722; 2.633; 2.501; 2.42; 2.292; 9 2.101; 1.92; 1.879; 1.723
Portlandite	4.928; 2.633; 1.967; 1.799; 1.689
Calcite	3.48; 2.912; 2.141; 2.209
Hydrohalite	3.867; 3.302; 1.998

## Data Availability

The original contributions presented in this study are included in the article. Further inquiries can be directed to the corresponding authors.
